# Two-Step Covalent Docking with Attracting Cavities

**DOI:** 10.1021/acs.jcim.3c01055

**Published:** 2023-12-04

**Authors:** Mathilde Goullieux, Vincent Zoete, Ute F. Röhrig

**Affiliations:** † SIB Swiss Institute of Bioinformatics, Molecular Modeling Group, CH-1015 Lausanne, Switzerland; ‡ Department of Oncology UNIL-CHUV, Lausanne University, Ludwig Institute for Cancer Research Lausanne Branch, CH-1066 Epalinges, Switzerland

## Abstract

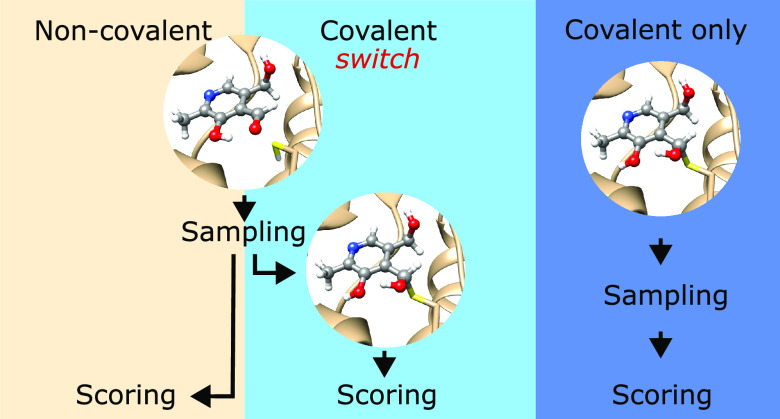

Due to their various advantages, interest in the development of covalent drugs has been renewed in the past few years. It is therefore important to accurately describe and predict their interactions with biological targets by computer-aided drug design tools such as docking algorithms. Here, we report a covalent docking procedure for our in-house docking code Attracting Cavities (AC), which mimics the two-step mechanism of covalent ligand binding. Ligand binding to the protein cavity is driven by nonbonded interactions, followed by the formation of a covalent bond between the ligand and the protein through a chemical reaction. To test the performance of this method, we developed a diverse, high-quality, openly accessible re-docking benchmark set of 95 covalent complexes bound by 8 chemical reactions to 5 different reactive amino acids. Combination with structures from previous studies resulted in a set of 304 complexes, on which AC obtained a success rate (rmsd ≤ 2 Å) of 78%, outperforming two state-of-the-art covalent docking codes, genetic optimization for ligand docking (GOLD (66%)) and AutoDock (AD (35%)). Using a more stringent success criterion (rmsd ≤ 1.5 Å), AC reached a success rate of 71 vs 55% for GOLD and 26% for AD. We additionally assessed the cross-docking performance of AC on a set of 76 covalent complexes of the SARS-CoV-2 main protease. On this challenging test set of mainly small and highly solvent-exposed ligands, AC yielded success rates of 58 and 28% for re-docking and cross-docking, respectively, compared to 45 and 17% for GOLD.

## Introduction

In the history of drug discovery, the development of covalent inhibitors was neglected for a long time due to safety concerns. This apprehension mostly came from studies revealing that certain compounds, such as bromobenzene or acetaminophen, can form reactive metabolites which covalently bind to liver proteins and cause hepatotoxicity.^1,2^ In spite of these concerns, covalent drugs such as aspirin, β-lactam antibiotics, or proton-pump inhibitors have proven to be safe and effective.^3,4^ Recent Food and Drug Administration approvals include a range of rationally designed covalent inhibitors in different indications such as cancer, viral diseases, and sickle-cell anemia.^5^ For example, the tyrosine kinase inhibitors Ibrutinib^6^ and Osimertinib^7^ are widely used to treat lymphocytic leukemia and lung cancer, respectively. The last few years have additionally witnessed various advances in the development of covalent ligands inhibiting the severe acute respiratory syndrome coronavirus 2 (SARS-CoV-2) main protease.^8^ These examples illustrate a renaissance in the development of covalent drugs in recent years.^3,9^ The strength of the covalent interaction between the ligand and the target reduces the off-rate and increases the residence time of the ligand in its target. Therefore, covalent drugs offer certain advantages over classical noncovalent ones. First, the dosage and frequency of treatment can be decreased, potentially reducing side effects.^10,11^ Second, shallow binding pockets, which might not be druggable with noncovalent ligands, can be targeted. As an example, the solvent-exposed K-Ras G12C mutated residue can be targeted by Sotorasib, a reactive Michael acceptor-carrier inhibitor.^12^ Third, covalent drugs may be able to bind to protein variants when noncovalent ones fail.^4,13,14^ For example, irreversible covalent inhibitors of the epidermal growth factor receptor can circumvent acquired resistance to gefitinib.^15^ However, they are, of course, very vulnerable to mutation of their covalently bound amino acid side chain.

Recent work has been devoted to reducing the safety liabilities of covalent drugs resulting from off-target binding by tuning ligands to specific binding sites. This can be achieved through developing targeted covalent inhibitors with high selectivity.^16−18^ The binding of a covalent drug usually consists in the reaction of an electrophilic warhead of the ligand with a nucleophilic side chain in the protein, such as acrylamides reacting with cysteines.^19^ By choosing specific chemical functions (warheads)^20^ and optimizing the combination of reactivity with target complementarity,^10,21,22^ it has become possible to tailor covalent drugs for specific targets, reducing safety concerns. For instance, this can be achieved through fragment-based drug discovery methods^23^ applied to covalent compounds. Innovative chemical biology tools for validating the activity, selectivity, and toxicity of covalent ligands have been developed over the past decade and provide a toolbox for charting new territories in drug design.^18^

During the last decades, advances in computing resources and methods have led to an increased utilization of computational techniques in medicinal chemistry.^24,25^ Understanding the interactions between ligands and proteins has become easier, faster, and cheaper thanks to structure-based computer-aided drug design (SB-CADD) tools. These include molecular docking algorithms used for predicting the binding mode of a ligand to its macromolecular target. The need for docking codes able to treat covalent inhibitors and to represent the formation of the covalent bond has been recognized.^26^ Therefore, popular codes such as AutoDock (AD),^27,28^ Glide (CovDock),^29,30^ FITTED,^31−34^ ICM-Pro,^35,36^ and genetic optimization for ligand docking (GOLD),^37−39^ now include a covalent docking option. Besides, other programs have been specifically developed for covalent docking, such as CovalentDock (built on AD),^40^ Cov_DOX,^41^ Covalent CDOCKER,^42^ WIDOCK,^43^ and HCovDock.^44^ Efforts have been undertaken to improve the simplicity and accessibility of computational screening for covalent ligands, for example, by creating freely available Web servers such as SCARdock.^45^ Some of these approaches have been successfully used to develop new hits by virtual screening or to refine the interactions of a ligand with its target.^46,47^ However, in a benchmarking study, it was found that the re-docking success rates of covalent docking codes reached only 40–60%,^48^ which leaves room for improvement.

The binding of a targeted covalent ligand to a protein occurs in two steps (eq 1)

- Step 1: formation of a noncovalent complex between the protein and the ligand in its prereactive topology, driven by electrostatics and van der Waals interactions,
- Step 2: chemical reaction, formation of the covalent bond via an exchange of electrons

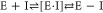
1with E: enzyme; I: inhibitor; and [E·I]: the transient noncovalent complex.

The first step is a reversible process, while the second step can be reversible or irreversible depending on the free energy profile of the reaction. The covalent docking algorithms of GOLD, ICM-Pro, and AD, as well as the dedicated covalent docking algorithms CovalentDock, COV_DOX, and HCovDock, do not consider this two-step mechanism and include the covalent bond between the ligand and the protein from the beginning, thereby restricting the sampling.^49^ On the other hand, the CovDock approach of Glide, FITTED, CDOCKER, and WIDOCK model the two reaction steps.

Quantum mechanical (QM) methods have also been investigated as a means to improve the docking score by better taking into consideration the formation of the covalent bond.^50^ For instance, the WIDOCK procedure is based on derived ligand reactivity against cysteine residues, either from experiments or from density functional theory calculations. These energies were then used to refine the scoring in a classical covalent docking algorithm.^43^ The method showed high sensitivity for retrieving active compounds and predicted seven human monoamine oxidase A inhibitors, which were experimentally confirmed.^43^ Cov_DOX, on the other hand, uses multiscale QM calculations directly in its docking algorithm with three levels of potential energy refinement. The authors report a re-docking success rate of 81% for a benchmark set^48,51^ of 405 covalent ligand–protein complexes.^41^ Taken together, these results suggest that QM methods are favorable for refining the description of covalent systems.

In the present study, we develop a classical covalent docking method mimicking the physical two-step mechanism of covalent binding (eq 1). It first freely samples noncovalently bound ligand poses, before forming the covalent link in situ, determining the postreactive stereochemistry of the complex on-the-fly. We implement this covalent docking method in our in-house docking program Attracting Cavities (AC), which was shown to yield high success rates for noncovalent docking benchmark sets based on its force field (FF)-based scoring function and thorough sampling procedure.^52,53^

As we were dissatisfied with the quality and diversity of available covalent structure re-docking benchmark sets, we developed a new high-quality set of 95 covalent complexes to assess the performance of our algorithm. The resulting dataset includes five reactive amino acid side chains and eight chemical reactions. All input files, including 3D-structures and FF parameters of this benchmark set, are publicly available. We compared the results of AC with two state-of-the-art covalent docking algorithms, GOLD^38^ and AD.^28^

## Methods

### Covalent Docking Procedure of AC

The most recent version of AC^53^ was used to implement the covalent docking algorithm. In AC, the rough energy landscape of the target protein is replaced by a smooth, attractive energy landscape during sampling. The sampling space is generated by using virtual attracting and electrostatic cloud points placed in concave regions of the protein surface. A threshold parameter *N*_Thr_ can be tuned to restrict the attracting points to deep binding clefts or to extend them also to shallower regions.^54^ The algorithm generates initial ligand conformations by placing, rotating, and minimizing the ligand inside this mold of the protein surface. The sampling procedure can be enhanced by applying small random variations to the initial conditions and using multiple random initial conditions (RIC).^53^ The sampling stage is followed by a pose refinement stage, in which the protein is reintroduced. Geometry optimizations are first performed with a soft-core potential, followed by the standard potential. Finally, the refined poses are scored by the CHARMM FF^55−58^ terms and the fast analytical continuum treatment of solvation (FACTS) model.^59^

**Figure 1:**
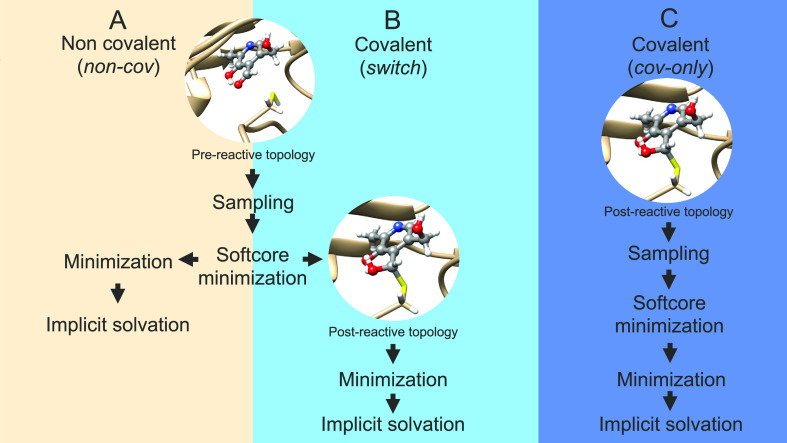
Different docking procedures are available in AC. (A) Noncovalent docking (*non-cov*); (B) covalent docking with a switch from the prereactive to the postreactive topology of the ligand (*switch*); and (C) covalent docking starting from the postreactive topology of the ligand (*cov-only*).

To mimic the biological binding process of a covalent ligand (eq 1), we implemented a two-step covalent docking method (Figure 1B) in AC. Sampling is performed with the noncovalent prereactive topology of the ligand (first stage of eq 1), while pose-refinement and scoring are done with the postreactive topology, which includes the covalent bond with the protein. We call this method the *switch* method (Figure 1B) and compare its performance to results obtained with purely noncovalent docking (*non-cov*, Figure 1A) and with purely covalent docking, starting directly from the postreactive topology of the ligand (*cov-only*, Figure 1C). In the switch method, poses obtained from noncovalent sampling can be filtered by the distance between the reactive sites of the ligand and the protein to account for the fact that covalent bond formation is unlikely to occur when the reactive sites are far from each other.

### Benchmark Sets

Different benchmark sets were developed to assess the performance of covalent docking algorithms. The earliest example was the set of 76 complexes used for the assessment of CovalentDock,^40^ consisting of 63 β-lactam ligands bound to Ser and 13 Michael acceptors bound to Cys (CS76). A set of 38 complexes (CS38) was used to assess Glide/CovDock, consisting of 29 covalent complexes bound to Cys and 9 to Ser.^30^ A larger set of 207 Cys-bound complexes (C207) based on published quality criteria^60^ was developed to compare the performance of different docking tools.^48^ The “benchmark data for covalent docking evaluation” (BCDE) set^51^ consists of 330 covalent complexes with 245 Cys and 85 Ser side chains. The addition of the C207 and BCDE sets and the removal of 35 cases for quality reasons led to the set of 405 covalent complexes used for the evaluation of Cov_DOX (CS405),^41^ consisting of 330 Cys-bound and 75 Ser-bound complexes formed by 8 reactions. For evaluation of the covalent docking routine of FITTED, the C207 set and 73 additional complexes bound to Cys (28 complexes), Ser (16), Lys (10), His (8), Tyr (6), Glu (2), Asp (2), and Met (1) were used (Var280).^34^ A few Web sites have been devoted to covalent complex databases, such as the cBinderDB^61^ (no longer accessible) and the CovalentInDB.^62^ Recent Web sites entirely based on structural data from the protein data bank include the CovPDB server^63^ and the CovBinderInPDB.^64^ We downloaded the data from both servers on February 1, 2023, yielding 2261 complexes from CovPDB (CPDB) and 3555 complexes from CovBinderInPDB (CBIP). We analyzed the content and quality of these 7 sets (Table 1). As most docking codes have previously been tested and compared on benchmark sets containing solely serines and cysteines,^41,48^ and as we noted a strong dependence of docking success on the quality of experimental data,^53^ we created in the following our own benchmark set (CSKDE95), optimized for structure diversity and quality.

**Table 1:** Quality Assessment of Covalent Complex Collections^a^

		overall structure quality				ligand properties						ligand drug-likeness					
Set	Tot	Data	Res	DPI	Pass	Lig	NoMiss	NoAlt	1Link	Bfct	Pass	MW	DOF	Ghose	Lip	Pass	Perc [%]
CS76^40^	76	62	64	61	53	53	49	45	53	53	41	40	41	39	41	39	51.3
CS38^30^	38	37	22	22	21	19	19	19	19	19	19	14	19	14	19	14	36.8
C207^48^	207	188	207	189	174	173	164	168	169	169	154	113	146	107	153	106	51.2
BCDE^51^	330	295	288	270	248	233	223	223	230	227	204	155	195	143	201	133	40.3
CS405^41^	405	363	368	339	311	298	282	283	294	292	259	191	245	177	258	169	41.7
Var280^34^	280	244	268	240	219	215	202	209	211	211	189	142	180	135	188	132	47.1
CSKDE95	95	95	95	95	95	95	95	95	95	95	95	95	95	95	95	95	100.0
CPDB^63^	2261	2068	2261	2023	1947	1781	1694	1657	1736	1762	1494	1284	1425	1083	1464	836	37.0
CBIP^64^	3555	3179	2833	2731	2399	2378	2280	2175	2232	2341	1900	1640	1815	1338	1863	1079	30.4

a Filtering was done in three successive steps: (1) overall structure quality, (2) ligand properties, and (3) ligand drug-likeness, and only complexes passing a step were considered in the next one. Set: name/abbreviation of the collection. Tot: total number of complexes. Data: X-ray structures with the available electron density. Res: resolution ≤ 2.5 Å. DPI: DPI ≤ 0.5 Å. Pass: number of complexes passing all filters of a step. Lig: covalently bound small-molecule ligand present. NoMiss: no missing ligand atoms. NoAlt: no alternate ligand conformations. 1Link: exactly one protein–ligand bond. Bfct: *B*-factor ≤ 80 Å^2^. MW: molecular weight 50–500 g/mol. DOF: less than 18 rotatable dihedrals. Ghose: respect at least 3 Ghose filters. Lip: respect at least 3 Lipinski rules. Perc: percentage of the total set that passes quality filters.

### CSKDE95 Benchmark Set

We applied the following quality filters to the structures available in the protein data bank (PDB)^65^ on January 13, 2021:

- X-ray structure with available electron density
- resolution ≤ 2.5 Å
- diffraction-component precision index (DPI)^66−68^ ≤ 0.5 Å
- contains a covalently bound small-molecule ligand (excluding, e.g., sugars and cofactors)
- exactly one covalent protein–ligand bond per ligand
- average *B*-factor of ligand ≤ 80 Å^2^
- no missing ligand atoms
- no alternative ligand conformations

The complexes were additionally analyzed with respect to the ligand properties, which were evaluated with SwissADME, a free web tool to evaluate the pharmacokinetics and drug-likeness of small molecules,^69^ by applying the following filters:

- molecular weight in the range [50 Da; 500 Da]
- less than 18 rotatable bonds
- respect at least 3 out of 4 Ghose filters^70^
- respect at least 3 out of 4 Lipinski rules^71^

Additionally, we requested that the ligand contains only elements C, O, N, H, S, F, Cl, and Br. Phosphorus, silicon, and iodine could be allowed, while boron and selenium needed to be excluded as they are not parametrized in the MMFF-like FF used for small-molecule modeling in AC.^54,72^ We applied the same quality filters to all seven previously published test sets and collections of covalent complexes. The results (Table 1) show that only 30 to 50% of the complexes included in previous benchmark sets pass our filters and are therefore suited for benchmarking docking algorithms.

Subsequently, we identified the chemical reaction occurring between the ligand and the reactive protein side chain for each complex. Complexes for which the reaction could not be identified due to a lack of information in the structure and the corresponding publication were excluded. For automated ligand FF generation purposes,^72^ we defined 8 reaction classes (Supporting Information, Figure S5), namely, (1) addition on carbonyl, (2) addition on nitrile, (3) addition on Michael acceptor, (4) nucleophilic substitution, (5) 3-ring opening (aziridine/epoxide), (6) disulfide formation (Cys only), (7) β- or γ-lactam opening (Ser only), and (8) imine formation (Lys only). Contrarily to most existing benchmark sets containing only cysteines and serines, we obtained valid complexes for five reactive protein side chains, namely, Cys, Ser, Lys, Asp, and Glu.

We chose to retain 10 complexes per reaction and reactive protein side chain, favoring diverse ligands and diverse active site structures, which we inspected visually. Applying this procedure, we obtained a total of 95 high-quality and diverse complex structures for the CSKDE95 benchmark set. In Figure S5, we display the pre- and postreactive chemical structures of the ligand for each chemical reaction of the benchmark set.

Apart from using CSKDE95 to develop our docking code, we additionally evaluated its performance on the CS405 set used by Wei et al.,^41^ which is the largest previously published benchmark set. However, for quality reasons, we considered only cases that passed our structure and ligand quality filters (259 cases; see Table 1). Additionally, we removed 4 cases with boron or phosphorus atoms in the ligand and 11 cases with reaction mechanisms that were either not clear or not implemented in our setup pipeline, leaving a total of 244 complexes. This set (CS244) is composed of 204 Cys and 39 Ser complexes. For comparison of AC with GOLD and AD, we used the combination of the CSKDE95 and CS244 sets, which resulted in 304 complexes (CSKDE304), considering the overlap of 35 complexes between the two benchmark sets.

### Additional Experimental Structure Assessment

To further assess the quality of the employed structural data, we calculated the molecular electron density score for individual atoms (EDIAm) for each ligand.^73^ We previously found that a ligand EDIAm value of 0.4 is sufficient to yield reasonable reference structures for docking.^53^ Calculation of the EDIAm value failed for a number of cases either due to a lack of experimental data or a mistreatment of crystal symmetry: CSKDE95 (11 cases), CS244 (14 cases), CS405 (76 cases), and SARS-MP-76 (45 cases). For 44 cases of the SARS-MP-76 set,^74^ the authors attributed a degree of confidence to the quality of the corresponding structure (Supporting Information, Table S5), which we used instead of the EDIAm value when it was not available. We also assessed, over all structures, whether the ligand of interest forms nonbonded interactions with a symmetry-related copy of the complex, which would be present in the experimental structure but absent in the docking calculations. To this end, we used the crystal contacts (CC) tool of UCSF Chimera^75^ with a cutoff length of 4.5 Å. Additionally, the portion of buried surface area of the ligands was calculated with CHARMM,^76^ using a probe radius of 1.4 Å. The results of this analysis (Tables S3–S5) and a comprehensive analysis of the structural properties of the benchmark sets are available in the Supporting Information.

### Protein and Ligand Structure Preparation

Each complex was visually examined with UCSF Chimera.^75^ Crystallization agents, uncoordinated ions, and solvent molecules were deleted using its DockPrep functionality. Incomplete protein side chains were added using the Dunbrack rotamer library.^77^ Histidines were protonated according to their environment, and missing hydrogen atoms were added using the HBUILD^78^ command of CHARMM.^76^ Protein chains situated further than 7 Å from the ligand were removed. When several copies of the ligand were present, we docked the one of chain A and kept all other copies in the complex fixed during docking.

UCSF Chimera was used to deduce bond orders and to add hydrogen atoms to each ligand based on its 3D coordinates. Ligand topologies were manually checked, corrected where necessary, and saved in mol2 format. SwissParam^54,72^ was used to generate the ligand FF topologies and parameters based on the Merck molecular FF.^79^ The latest version of SwissParam^72^ allows the parametrization of both pre- and postreactive topologies of a ligand starting from either form. Details of the procedure are given in the Supporting Information of ref (72).

In order to obtain a random initial conformation for each ligand, 10 random conformers were generated with Open Babel.^80^ The one with the highest rmsd was selected, rotated, submitted to a short molecular dynamics simulation at high temperature, minimized, and placed in the ligand binding site. Unless otherwise specified, all dockings started from randomized ligand conformations.

All complexes were described with the CHARMM36 FF^57,58^ and minimized with 100 steps of steepest descent and 200 steps of adopted basis Newton–Raphson to remove possible clashes. During the minimization, a constraint of 5 kcal/mol/Å^2^ was applied to all heavy atoms toward their experimental coordinates, and the structure was solvated with the FACTS model.^59^ Structures used for cross-docking were not minimized to avoid structural bias.

All calculations were carried out on a single AMD EPYC 7443 3.34 GHz CPU for AC and AD, and a single Intel i7-11700 2.50 GHz CPU was used for GOLD.

### Docking with AC

The cubic search box had an edge length of 20 Å and was centered on the center of mass of the ligand in the corresponding experimental structure. All dockings were performed with AC coupled to the CHARMM program,^76^ version 44b1. Poses were scored with the AC scoring function, given by the sum of the CHARMM total energy^57,58^ and the FACTS energy.^59^

In order to investigate the sampling power of AC, different sampling parameters (SP) were varied with the *switch* method (Figure 1B), namely, the initial rotational angle of the ligand, the concavity parameter *N*_Thr_, and the number of RIC. The attractive points were also used as ligand placement points for the initial sampling step. Electrostatic points were used for all of the docking runs.

We then selected the optimal parameters for each benchmark set and used them to compare the docking performance of the *non-cov*, the *switch*, and the *cov-only* methods (Figure 1). We also evaluated the use of a distance filter between the reactive atoms by using cutoffs of 5 and 10 Å for covalent docking with the *switch* method. Finally, the *switch* method was compared to the GOLD and AD covalent docking procedures.

### Docking with GOLD

**Figure 2:**
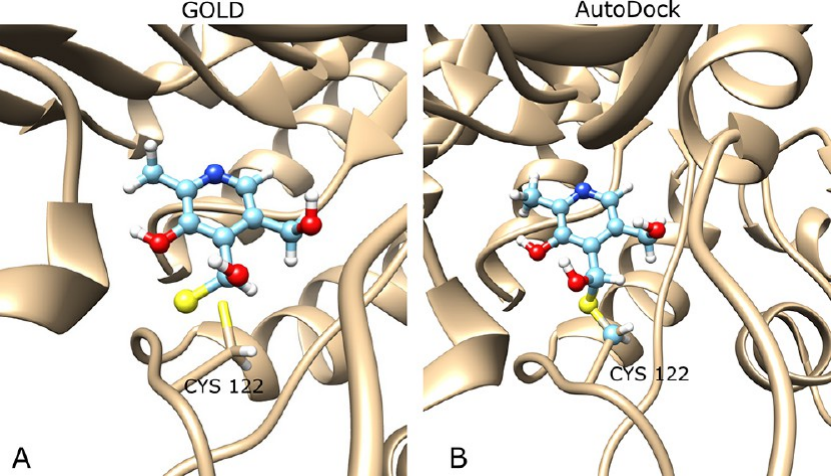
Example of link atom definition for the reaction of an aldehyde with a cysteine side chain (PDB ID 1td2). The ligand is depicted in ball and stick representation (light blue) and the protein and its docking site (Cys122, chain A) in stick representation (tan). (A) For GOLD, the link atom is the sulfur atom, present both in the ligand and the protein. No alignment is performed before the docking procedure. (B) For AD, the S and C_β_ atoms of Cys are added to the ligand for alignment with the protein docking site prior to docking.

We used the GOLD^37−39^ code, version 2022.2.0, from the Cambridge Crystallographic Data Centre. To maintain consistency with the volume of the cubic search box with a 20 Å edge length in AC, we used a spherical search space with a 12.4 Å radius. The center of the search space was defined as the center of mass of the native ligand pose. For covalent docking with GOLD, by default, a link atom corresponding to the reactive atom of the protein should be added to the ligand.^37^ The same (deprotonated) atom is also present in the protein. In Figure 2A, for instance, the link atom of the protein is the deprotonated reactive sulfur of the cysteine, and the ligand link atom is the sulfur atom added to the postreactive topology of the ligand. These two linkers are matched during the docking. Alternatively, it is possible to use the Cα atom of the reactive side chain as a link point, therefore taking side chain flexibility into account. This option was not tested in the present study. The “convert” option of gold_utils was used to convert protein structures to mol2 format from the corresponding CHARMM pdb file, and the atom types were checked and corrected by the check_mol2 script. Prior to docking, the ligand structures in mol2 format were minimized with the GOLD conformer_generator.

During docking, three different fitness functions were used to evaluate the performance of the code, GoldScore (GS),^37^ ChemScore (CS),^38^ and the piecewise linear potential (PLP).^81^ The rmsd tolerance for pose clustering was set to 2.0 Å, and by default, 100 GA runs were carried out, without early termination. An additional calculation with the PLP fitness function and 1000 GA runs was carried out to investigate the influence of increasing the sampling.

### Docking with AD

AD version 4^27^ is an open source and freely accessible docking tool, in which two different covalent docking procedures have been implemented.^28^ We chose the flexible side chain method for our study, as it was shown to perform better.^28^ The use of this method requires the addition of two link atoms from the target residue to the ligand. The ADs tool (ADT) suite^27^ was used to superimpose the two linkers placed in the ligand with their counterparts in the protein structure, allowing the creation of the covalent bond between the two molecules prior to docking. An example of such an aligned structure is presented in Figure 2B. Following the alignment procedure, ADT was used to calculate Gasteiger charges^82^ and to generate PDBQT files of the rigid and flexible parts of the complex. During the docking, AD treats the protein and the backbone of its reactive site as rigid and fixes the C_α_ and C_β_ atoms. The ligand is considered as a fully flexible extension of the protein reactive site. The Lamarckian Genetic Algorithm was used to perform the dockings, and all remaining parameters were set to their default values. The center of the cubic search space with an edge length of 20 Å was defined as the center of mass of the ligand. The five complexes with reactive Asp and Glu side chains of the CSKDE95 benchmark could not be docked with AD as these residues are not implemented in the alignment procedure of ADT.

### SARS-CoV-2 Main Protease Cross-Docking Dataset

According to the covPDB^63^ database, the SARS-CoV-2 main protease is the protein with the largest number of covalent ligand complexes in the PDB at present, with 81 unique structures, all ligands bound to Cys145. Application of our quality filters resulted in 78 structures, and the exclusion of two cases with reactions not implemented in the parametrization tools led to a set of 76 complexes (SARS-MP-76). As the target structure for cross-docking, we selected a complex presenting a wide, open cavity (PDB ID 7c7p). A detailed analysis of the properties of the SARS-MP-76 set can be found in the Supporting Information.

AC re-docking and cross-docking were performed using a sampling with 8 RIC, a rotational angle of 90°, and a concavity parameter *N*_Thr_ of 60. We performed cross-docking with a rigid protein, as well as two additional runs where (1) only the reactive residue Cys145, or (2) residues His41, Met49, Asn142, Gly143, Cys145, His163, His164, Met165, Glu166, Leu167, and Gln189 were flexible during the docking (Supporting Information, Figure S8). Re-docking and cross-docking with GOLD were carried out with the PLP scoring function. AD was excluded from this study due to the cumbersomeness of generating input structures for covalent cross-docking.

### Clustering and Docking Success Criteria

To analyze the results of AC, GOLD, and AD, we performed a clustering of the final poses. First, the poses were ordered by score and the DockRMSD algorithm, which accounts for the symmetry in a molecule,^83^ was used to calculate pairwise rmsd values. The docking pose with the best score was selected as the reference of the first cluster, and any pose with a rmsd below 2 Å to this reference was assigned to the same cluster. The next unclassified pose with the lowest score was chosen as the reference for the second cluster, and similar poses were added to the same cluster. This iterative clustering was performed for a maximum of 50 clusters with 8 poses each, additional poses with higher scores being discarded.

DockRMSD was also used to calculate the rmsd values between all docking poses and the native ligand conformation. A success was defined as finding a given pose with a rmsd value lower than a certain threshold. Three different threshold values were used: (i) 1.0 Å (Best-1.0); (ii) 1.5 Å (Best-1.5); and (iii) 2.0 Å (Best-2.0). We also assessed if at least one pose (iv) of the first cluster (Cluster1); (v) of the first five clusters (Cluster1–5); and finally (vi) among all poses (All) had a rmsd below 2.0 Å with respect to the native pose.

We also analyzed scoring and sampling failures. A scoring failure implies that the native pose does not have the best docking score, while a sampling failure implies that the native pose has the best score but is not sampled during a docking run. This definition provides a lower limit to the number of scoring failures because a scoring failure can only be detected if a pose with a lower score than the native pose is sampled during the docking run. All failures not attributed to scoring failures are classified as sampling failures, but they could, in fact, also be undetected scoring failures.

Scoring failures were defined based on the difference between the score of the highest ranked docking pose and the score of the experimental ligand structure. Rescoring was done with the RESCORE flag in GOLD and the pdbe command in AD. As done before for AC,^53^ we relaxed the failure criteria slightly and defined a scoring failure when (i) the rmsd of the highest ranked docking pose was >3.0 Å, and (ii) its score was lower than the score of the experimental pose by at least 1.0 [D], where [D] corresponds to kcal/mol for AD and AC and is dimensionless for GOLD.

## Results and Discussion

### Performance of the *Switch* Method in AC

The sampling level of AC is determined by different parameters, such as the concavity parameter *N*_Thr_, the initial ligand rotation angle, and the number of RIC.^53^ Their values can be optimized according to the properties of the target of interest, for example, depending on the concavity of the active site or the number of rotatable dihedrals of the ligand. Here, we performed an analysis of the influence of the concavity parameter *N*_Thr_ on the docking results performed on the CSKDE95 and the CS244 datasets (see Supporting Information), which demonstrated only a minor influence on the docking results. Therefore, we selected a single set of SP for the purpose of this study, namely, a concavity parameter *N*_Thr_ of 60, a rotational angle of 90°, and 8 RIC.

**Figure 3:**
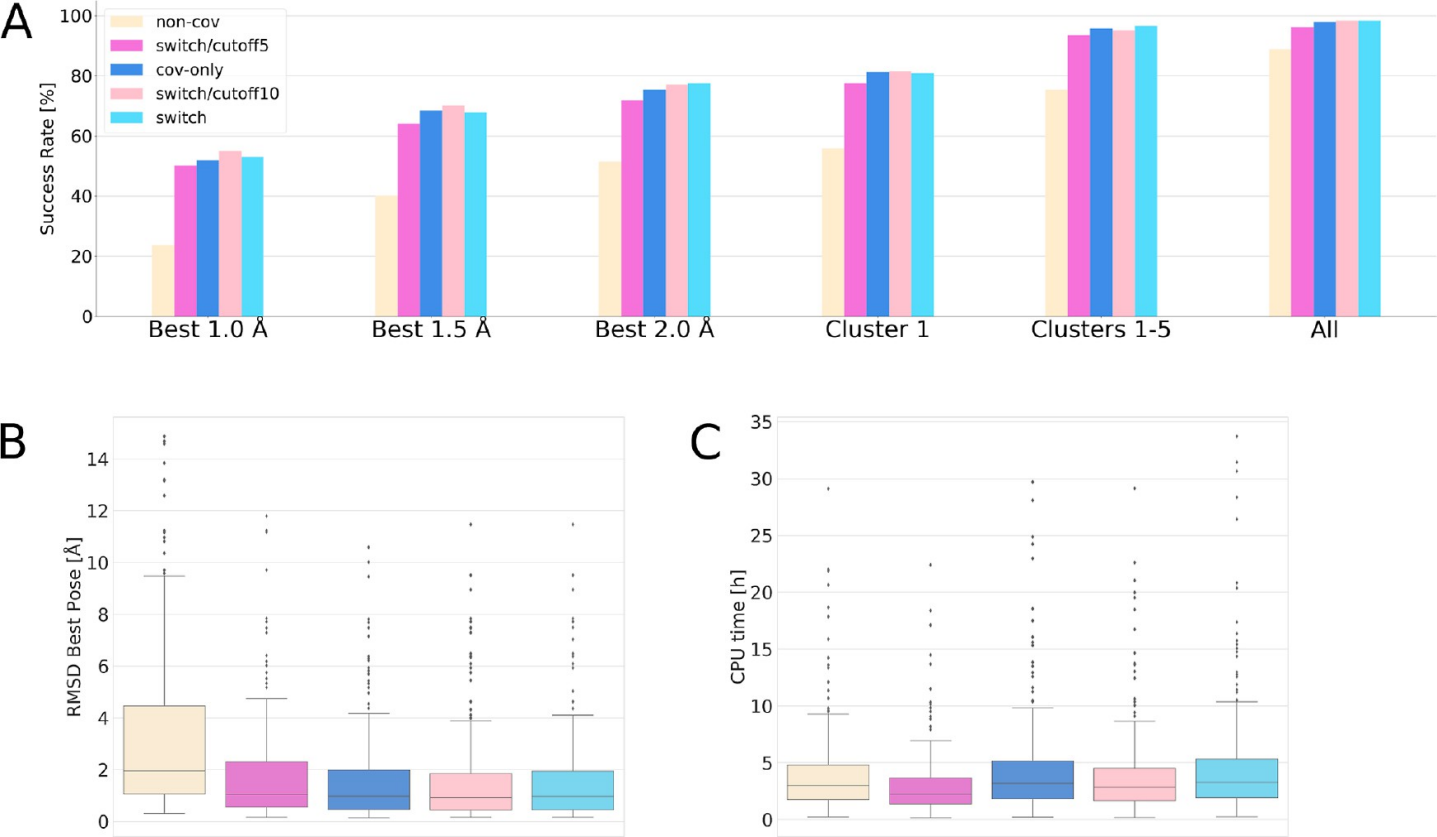
Re-docking results of AC on the CSKDE304 dataset using the *cov-only*, *switch*, and *non-cov* algorithms. For the switch method, 3 values for the distance filter were used (5 Å, 10 Å, no cutoff). (A) Success rates. (B) rmsd of the best pose. (C) CPU time. Numerical values given in Table S6.

In the following, we evaluated the docking success rates obtained (1) with the noncovalent (*non-cov*) procedure of AC, (2) when sampling and scoring with the postreactive topology (*cov-only*), and (3) using the *switch* procedure. The results demonstrate that using the *non-cov* method gives substantially lower success rates than the two other methods (Figure 3), due to the neglect of the chemical reaction leading to the covalent reference complex. However, the success rate obtained with this method (51%) remains comparable to the performance of some covalent docking codes.^48^ From Figure 3A, it is apparent that the success of obtaining a pose with a rmsd below 2 Å among all poses (all) is high with all three methods, indicating that the sampling procedure is effective. Using the *cov-only* method leads to an increase of success rate of 24% (Supporting Information, Table S6) due to the substantial improvement in the scoring, taking into account the formation of the covalent bond. The success rate can be further increased by 3% when the *switch* procedure is used, which allows for a better sampling of the first step of the ligand recognition process by the protein target. This points to the prevalence of nonbonded interactions during the sampling phase, before the formation of the covalent bond, and supports the idea that covalent docking can profit from reproducing this first step of ligand binding.

Using a distance cutoff between reactive sites of 10 Å to filter poses before covalent docking reduces the average computational time by 16% without a significant reduction in success rates (Supporting Information, Table S6). The success rates are slightly poorer (6%) when using a distance cutoff of 5 Å, but the corresponding decrease in computational time is more important, at roughly 33%. These results demonstrate the usefulness of this option for decreasing CPU times.

### Comparison of AC with GOLD and AD

To compare the covalent docking performance of AC to that of commonly used programs, we carried out covalent docking with AD and GOLD, using the combined CSKDE304 set.

**Figure 4:**
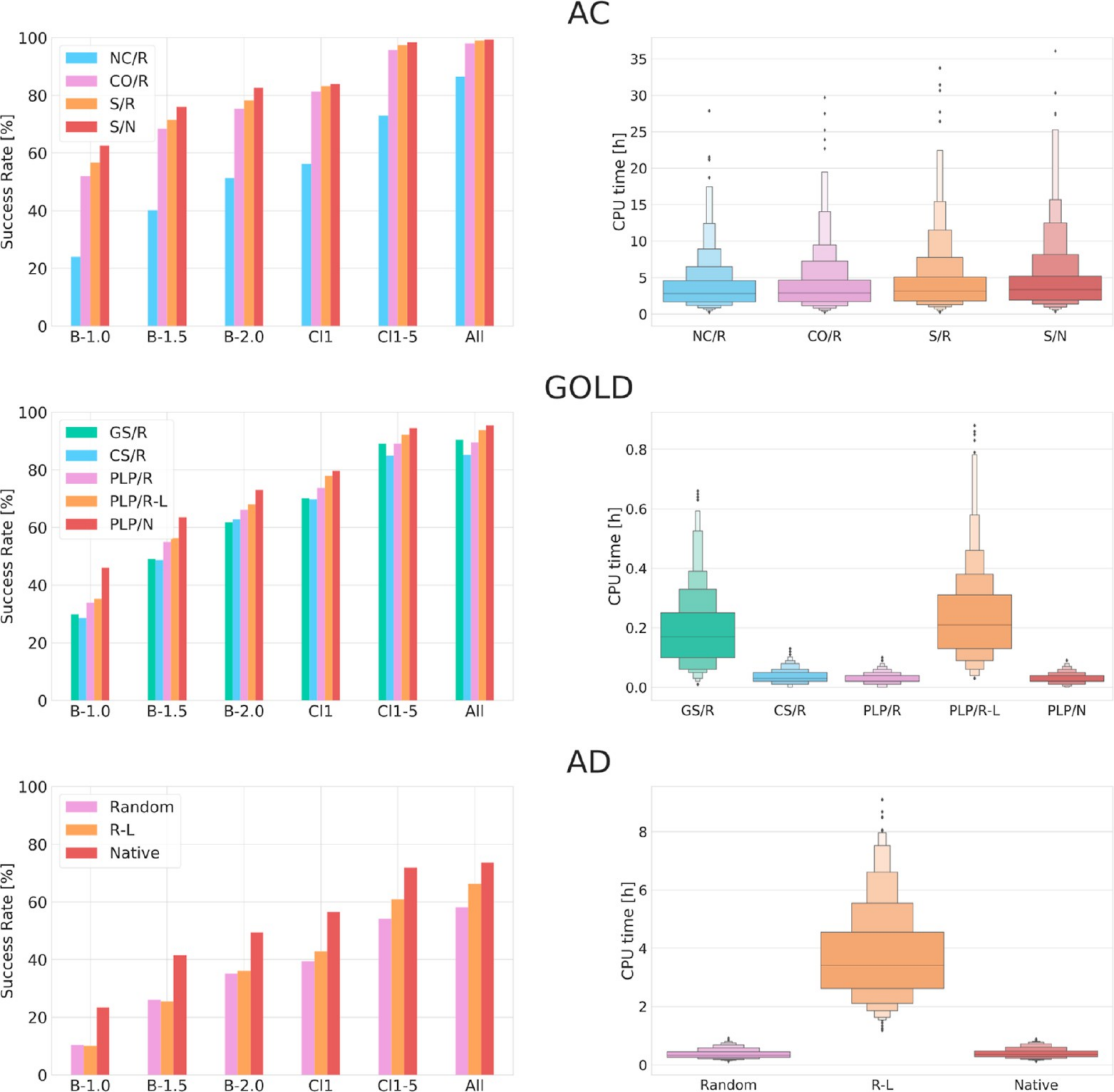
Comparison of re-docking results obtained with AC, GOLD, and AD on the CSKDE304 set. Left: success rates and right: CPU times. For AC: docking with *non-cov* (NC), *cov-only* (CO), and *switch* (S) methods; For GOLD: docking with Goldscore (GS), Chemscore (CS), and PLP (PLP) scoring functions. All dockings were performed by starting from the random (R) and native (N) conformations of the ligand. For GOLD and AD, long dockings (L) are performed with 1000 poses. Numerical values given in Supporting Information, Table S7.

AD gives rather poor results compared to GOLD and AC and reaches a success rate of 35% for re-docking from a randomized ligand conformation (Best-2.0, Figure 4). Additionally, the success rate of finding a good pose among all poses is only 58%, hinting at a sampling issue. Indeed, visual inspection of all generated poses suggests low structural diversity. However, increasing the sampling by using 1000 GA runs increases the success rate by only 1% (Supporting Information, Table S7). These results are lower than what has been reported by Scarpino et al.,^48^ who found a success rate of 55% for covalent docking with AD, and may be due to different procedures to generate randomized initial ligand conformations. We obtain a success rate similar to the literature value (49%) only when starting from the native ligand conformation.

GOLD performs much better than AD in our hands and reaches a success rate for the best pose (rmsd ≤ 2 Å) of 66% when using the PLP scoring function, 62% with GS, and 63% with CS (Figure 4). Increasing the sampling from 100 poses to 1000 poses with the PLP scoring function increases the success rate by 2%. CPU times are very low when compared to those of AD and AC (Supporting Information, Table S7), especially when using the PLP scoring function, with which an average docking takes less than 2 min. The success rate of finding a good pose among all final poses is generally larger than 90%, suggesting a good sampling.

AC clearly outperforms the two other docking codes for covalent re-docking (Figure 4). Using the *cov-only* method, which considers the covalent link from the docking initialization—similarly to AD and GOLD—gives success rates approximately 10% higher than GOLD/PLP, while the *switch* method gives an even superior success rate (78%, Supporting Information, Table S7). The success rate of 51% obtained with the *non-cov* method of AC is comparable with the ones obtained with other docking codes using their covalent docking feature.^48^ AC also shows the smallest drop in success rate between dockings starting from the native or a random conformation of the ligand (5%), as compared to GOLD (7%) and AD (14%), highlighting its good sampling capabilities (Supporting Information, Table S7). As a drawback, this sampling comes at a relatively high computational price.

We studied the influence of the complex properties on the docking performance by assessing the rmsd of the best pose with regard to ligand flexibility, buriedness, and structural quality, as well as the number of crystal contacts (CC) it forms. Small rigid ligands show a lower median rmsd than ligands with a higher number of rotatable dihedrals (Figure S6A). As expected from our previous study,^53^ highly buried ligands without CC resulted in lower median rmsd values than ligands with high solvent accessibility or forming at least one CC (Figure S7A,B). Removal of all complexes from the CSKDE304 set where the ligand forms CC leaves 212 cases (CSKDE212) and improves the success rate (Best-2.0) of AC from 78 to 81%, of GOLD from 66 to 71%, and of AD from 35 to 40% (Supporting Information, Table S7). These findings suggest that structures containing CC should either be excluded from benchmark sets or the copies of the protein making CC with the ligand should be included in the receptor structure for docking.

The results of the classification of scoring and sampling failures (Supporting Information and Table S8) show indeed that AD presents almost exclusively sampling failures, so little can be said about its scoring failures, while for AC and GOLD, about one-third of failures can be attributed to scoring failures. For all docking codes, highly solvent-exposed ligands are more enriched among scoring failures than among sampling failures. Ligands with CC are mainly enriched among the scoring failures of AC, showing the sensitivity of AC to this issue.

In this study, numerous efforts were made to filter benchmark structures based on their quality, but the electron density support of the ligand conformation was not used during filtering. To account for this property, we analyzed the best pose rmsd with respect to the ligand EDIAm value,^73^ classifying them into low, medium, high, and unknown quality, the latter accounting for cases where the EDIAm calculation failed. The analysis (Supporting Information, Figure S7C) demonstrates that lower-quality structures coincide with worse docking results, suggesting that confidence in the structure, specifically in the ligand binding site, should be assessed before performing docking benchmark studies. Otherwise, perceived docking failures may be due to inaccuracies in the experimental reference model.

### Re-Docking and Cross-Docking to SARS-CoV-2 Main Protease

**Figure 5:**
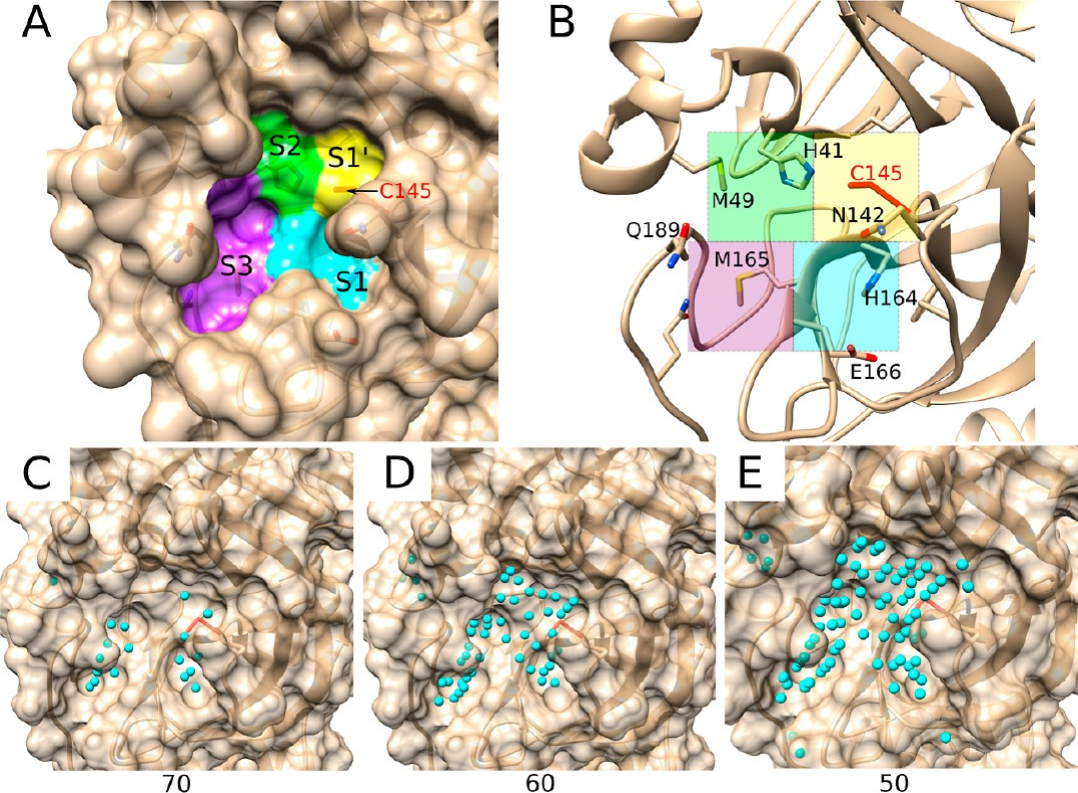
Structure of the SARS-CoV-2 main protease. (A,B) Active site structure (PDB ID 5rej) with its 4 subpockets^74^ and the reactive Cys145 residue highlighted. (C–E) Surface representation (PDB ID 7c7p), showing AC attractive points (cyan) with *N*_Thr_ = 70, 60, and 50, respectively.

We compared the re-docking and cross-docking performance of AC and GOLD on the SARS-MP-76 benchmark set. This set is challenging for docking as a large part of its ligands are very solvent exposed (Supporting Information and Figure S2G). For AC, we first assessed different values of the concavity parameter *N*_Thr_ for the placement of attractive and initial ligand placement points during the sampling phase. Using the target structure of the cross-docking calculations (PDB ID 7c7p), we obtained 25 points with an *N*_Thr_ value of 70 (Figure 5C), 45 points with a value of 60 (Figure 5D), and 84 points with a value of 50 (Figure 5E). From visual inspection, it is evident that a value of 70 gave an insufficient number of attractive/placement points, not covering the entirety of the cavity and its four subpockets. A value of 60 seemed to be a good compromise for faithfully mimicking the shape of the binding pocket at a reasonable computational time. In the following, we used this value, which was also used for all previously reported re-docking calculations.

**Figure 6:**
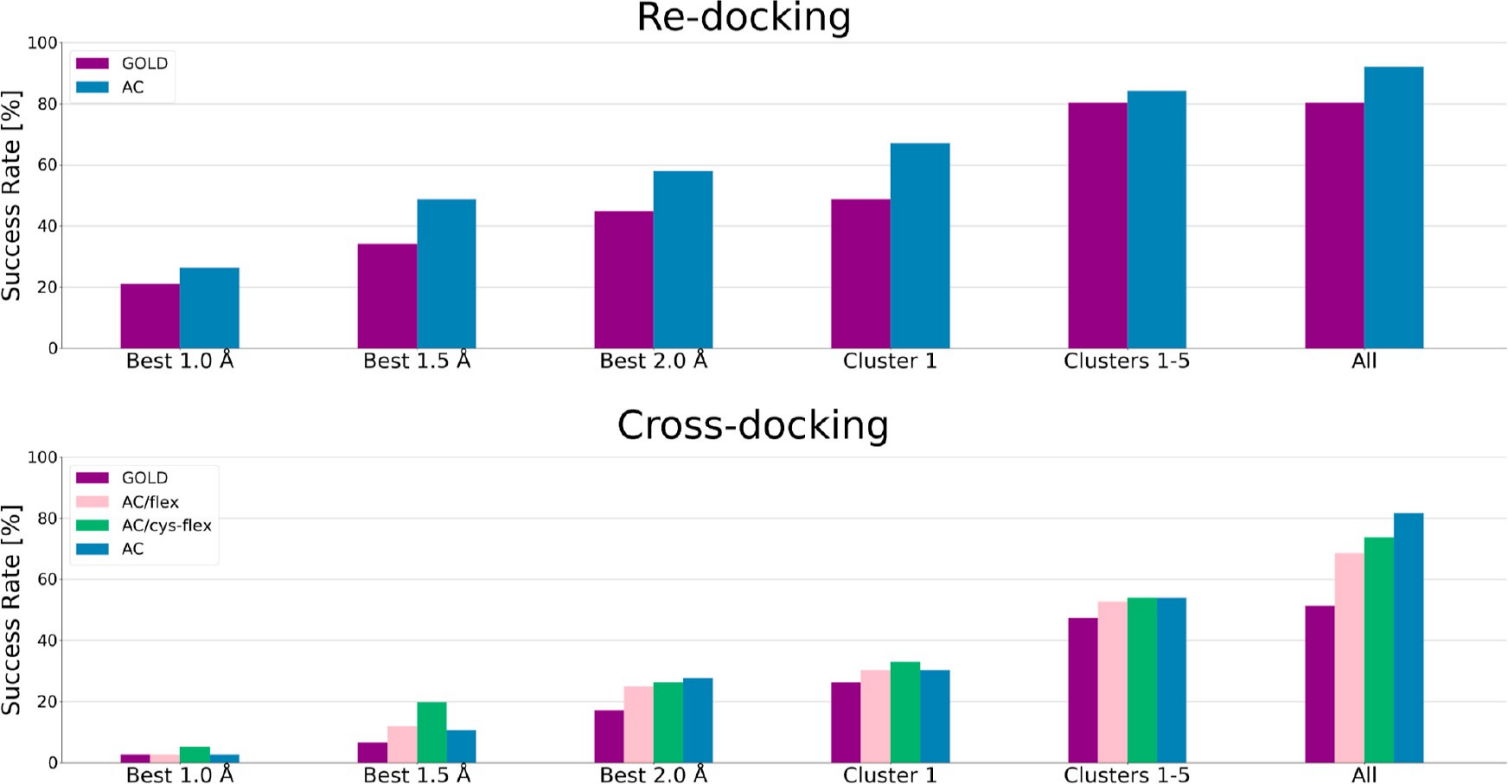
AC and GOLD results on the SARS-MP-76 benchmark set with *switch* and PLP methods, respectively. For AC, cross-docking with flexible residues (flex) and flexible sulfur on the cysteine (cys/flex) has been tested (Supporting Information, Figure S8). Numerical values are given in Table S9.

The re-docking success rate of AC (58%, Figure 6) was low compared to the one obtained with CSKDE304 (78%), demonstrating the difficulty of docking the ligands in this shallow, open cavity (Figure 5). Inline with these results, GOLD only obtained a re-docking success rate of 45% (66% for CSKDE304). Removal of all cases with CC leaves a reduced dataset of 39 cases (SARS-MP-39) and increases the AC re-docking success rate from 58 to 64% and the one of GOLD from 45 to 51% (Supporting Information, Table S9).

The AC cross-docking success rate of 28% corresponds to a decrease of 30% as compared to the re-docking experiment, which is similar to the drop seen in the previous cross-docking result of AC^53^ and close to the drop of 28% from re-docking to cross-docking with GOLD (Supporting Information, Table S9). We additionally assessed the influence of a flexible protein environment for cross-docking with AC. Flexibility of just the reactive Cys145 residue or of all residues susceptible to generate clashes with any of the ligands (11 amino acids, Supporting Information and Figure S8) did not show a strong influence on the success rate (26 and 25%, respectively). This may be due to the fact that the cavity of the chosen target structure is wide open and does not generate many clashes with different ligands. Analysis of the rmsd value of the best pose versus ligand properties (Figure S9) demonstrates that, also for cross-docking, the presence of CC and the buriedness of the ligand have a high impact on the quality of the docking predictions.

## Conclusions

The class of covalent inhibitors has become a powerful asset to target specific proteins, and covalent docking algorithms are under development to improve the CADD of such compounds. In this study, we first created a diverse, manually curated, high-quality benchmark set of covalent ligand–protein complexes called CSKDE95. This open-access set provides a basis for benchmarking other covalent docking algorithms in the future.

We then used the CSKDE95 set, in addition to good-quality structures from a previously published set (CS244), to assess a new covalent docking method implemented in our in-house docking code AC. AC reached high success rates for re-docking from randomized ligand conformations, up to 81% on the complexes of the CSKDE95 set and 79% on the CS244 set. In agreement with earlier studies, the rmsd of the best pose was found to be significantly dependent on three factors. First, the prediction of the correct binding mode is dependent on the buriedness of the ligand. The more contacts the ligand makes with the protein, the easier the prediction. This remains true despite the covalent anchoring point present in covalent ligands. Second, the existence of CC in the experimental structure impedes the prediction of the native binding mode because these interactions are absent in the docking calculation. Third, the quality of the experimental data in the ligand binding site plays an important role as a poorly defined ligand conformation is more difficult to correctly predict than a well-defined conformation. This highlights the importance of using high-quality structures for benchmarking purposes. We tested several SP of AC and found that an initial ligand rotation of 90° in combination with 8 RIC and a concavity parameter (*N*_Thr_) of 60 worked well for all covalent benchmark sets.

Our covalent docking method (*switch*) follows the two-step mechanism of covalent inhibitors and was compared to noncovalent docking (*non-cov*) and covalent-only docking (*cov-only*). On the test set combining the CSKDE95 and CS244 sets (CSKDE304), the *switch* method performed better (success rate 78%) than *cov-only* (75%), and substantially better than *non-cov* (51%), demonstrating the advantage of focusing on noncovalent interactions in the sampling step and on the combination of noncovalent and covalent interactions in the scoring step. Using a distance cutoff to filter poses before covalent docking can save computational time for a small decrease in success rate. Using a cutoff of 5 (72%) and 10 Å (77%) decreased the CPU time by 33 and 16%, respectively.

We compared the re-docking performance of AC with the two state-of-the-art covalent docking algorithms of GOLD and AD (flexible side-chain method), using a combined set of 304 covalent complexes. With a success rate of 78%, AC clearly outperforms GOLD (66%) and AD (35%). When using a more stringent success criterion (best pose rmsd ≤ 1.5 Å), AC still reaches a success rate of 71% vs 55% for GOLD and 26% for AD.

Covalent docking with GOLD was fast and easy to setup, but special attention must be paid to the atom types in the protein and ligand structure input files. Covalent docking with AD, on the other hand, required a substantial time investment for generating high-quality input files. The covalent docking success rate we obtained with GOLD was better than the one reported in a comparative benchmark study (66 vs 53%), while the one for AD was substantially lower (35 vs 55%).^48^ As the input files used in that study are not available, we do not know the underlying reasons for these differences. Other covalent docking codes were not tested in this study so we cannot comment on their performance compared to AC.

We assessed the cross-docking performance of AC on an additional dataset gathering 76 structures of the SARS-CoV-2 main protease cocrystallized with diverse ligands. The dataset is challenging due to the high solvent exposure and low specificity of the ligands. In re-docking, AC reached a success rate of 58% versus 45% for GOLD. Cross-docking led to a drop in success rate of roughly 30% for both programs, which is in agreement with what was found in our previous study on noncovalent ligands.^53^ Leaving the reactive cysteine or a set of selected active site residues flexible did not improve the results.

In summary, our results highlight the value of the covalent docking method implemented in AC and its usefulness for drug design studies. Since AC is computationally too demanding for large scale screening, it will be most useful in lead optimization or fragment-based drug design settings. To increase its user friendliness, we created a tool to handle the setup of covalent complexes, which is available on the SwissParam 2023 Web server.^54,72^ In the near future, the AC docking algorithm will be made freely accessible through a new version of the SwissDock Web server.^84^

## Data Availability

The data used to generate the results of this manuscript (parameter files, topology files, input files, and output analysis scripts) are available on Zenodo (10.5281/zenodo.8407429). The covalent AC docking code, as well as the parameter and topology files will be made available through the SwissDock Web server (www.swissdock.ch)^84^ in the near future.

## Supporting Information Available

Software and procedures mentioned can be accessed on the following Web sites: ADT for generating Vina input files (https://autodocksuite.scripps.edu/adt/), CHARMM molecular simulation program (https://www.charmm.org), DockRMSD for rmsd calculation of symmetric molecules (https://zhanggroup.org/DockRMSD/), DPI calculator (http://users.abo.fi/mivainio/shaep/download.php), server to calculate EDIAm values (https://proteins.plus/), GOLD program (https://www.ccdc.cam.ac.uk/solutions/software/gold), Open Babel version 2.4.1 for ligand coordinate manipulation (https://openbabel.org/), SwissParam server for generating ligand FF for AC (https://www.swissparam.ch/), UCSF Chimera software for analysis and visualization (https://www.cgl.ucsf.edu/chimera),^75^ MarvinSketch 20.15, 2020, ChemAxon (http://www.chemaxon.com), for drawing, displaying, and characterizing chemical structures
Assessment of benchmark sets; analysis of AC sampling parameters; classification of benchmark sets by protein family; AC re-docking results with *switch* method; properties of the CSKDE95 set; properties of the CS244 set; properties of the SARS-MP-76 set; AC re-docking results with *non-cov*, *switch*, and *cov-only* methods; AC, GOLD, and AD re-docking results; analysis of AC, GOLD, and AD failures; AC and GOLD re-docking and cross-docking results; classification of benchmark sets by protein family; structural properties of benchmark sets; AC re-docking results with *switch* method; example of problematic complex; summary of chemical reactions; AC, AD, and GOLD re-docking analysis; definition of SARS-CoV-2 main protease flexible residues; AC and GOLD cross-docking analysis; and AC re-docking and cross-docking results (PDF)

## Abbreviations

AC: Attracting Cavities
AD: AutoDock
ADT: AutoDock Tools
CADD: computer-aided drug design
CC: crystal contacts
CS: ChemScore
DPI: diffraction-component precision index
EDIA: electron density support for individual atoms
EDIAm: EDIA for multiple atoms
FACTS: fast analytical continuum treatment of solvation
FF: force field
GA: genetic algorithm
GOLD: Genetic Optimization for Ligand Docking
GS: GoldScore
RIC: random initial conditions
rmsd: root-mean-square deviation
PLP: piecewise linear potential
SP: sampling parameters
