# Supplementary material for: Two-Step Covalent Docking with Attracting Cavities
Source: J Chem Inf Model. 2023 Dec 4;63(24):7847–59. doi: 10.1021/acs.jcim.3c01055 (PMC10751798; doi:10.1021/acs.jcim.3c01055)
Supplement: Supplementary file 1 — ci3c01055_si_001.pdf [file ci3c01055_si_001.pdf]

Supporting Information: Two-Step Covalent Docking with Attracting Cavities

Mathilde Goullieux,[†] Vincent Zoete,[†,‡] and Ute F. Röhrig[*,†]

†*SIB Swiss Institute of Bioinformatics, Molecular Modeling Group, CH-1015 Lausanne, Switzerland*

‡*Department of Oncology UNIL-CHUV, Lausanne University, Ludwig Institute for Cancer Research Lausanne Branch, CH-1066 Epalinges*

E-mail: ute.roehrig@sib.swiss

Contents

Assessment of Benchmark Sets **3**

Analysis of AC Sampling Parameters **7**

Supporting Tables **11**

Supporting Figures **30**

List of Tables

S1 Classification of benchmark sets by protein family (CSKDE95, CS244) . . . 4

S2 AC re-docking results with *switch* method (CSKDE95, CS244) 9

S3 Properties of the CSKDE95 set 11

S4 Properties of the CS244 set 15

S5 Properties of the SARS-MP-76 set 23

S6 AC re-docking results with *non-cov*, *switch*, and *cov-only* methods 26

S7 AC, AD, and GOLD re-docking results (CSKDE304, CSKDE212) 27

S8 Analysis of AC, AD, and GOLD docking failures (CSKDE304) 28

S9 AC and GOLD re-docking and cross-docking results (SARS-MP-76 and SARS-MP-39) 29

List of Figures

S1 Classification of benchmark sets by protein family (CSKDE95, CS244) . . . 5

S2 Structural properties of benchmark sets (CSKDE95, CS244, SARS-MP-76) . 6

S3 AC re-docking results with *switch* method (CSKDE95, CS244) 8

S4 Example of problematic complex (PDB ID 4i9o). 10

S5 Summary of chemical reactions 30

S6 AC, AD, and GOLD re-docking analysis A (CSKDE304) 31

S7 AC, AD, and GOLD re-docking analysis B (CSKDE304) 32

S8 Definition of SARS-CoV-2 main protease flexible residues 33

S9 AC and GOLD cross-docking analysis (SARS-MP-76) 34

S10 AC re-docking and cross-docking results (SARS-MP-76) 35

Assessment of Benchmark Sets

We assessed the structural properties of the benchmark sets used to evaluate the different docking algorithms selected for our study. In CSKDE95 the number of complexes per reactive protein side chain differs due to data availability. From our filtering procedure, we obtained suitable high-quality structures for cysteine (60 complexes), serine (20), lysine (10), aspartate (3), and glutamate (2), resulting in a total of 95 complexes of 69 different proteins. Even though CS244 contains more structures, it is less diverse because it is restricted to Cys (205) and Ser (39) reactive side chains.

Using the enzyme commission (EC) number and the UniProt ID,[1] we classified all complexes of CSKDE95 and CS244 by protein family. The distribution of complexes among families is similar in both benchmark sets (Figure S1), with a majority of hydrolases. These proteins are crucial in various biological processes, such as breaking down nutrients into smaller units for digestion. The transferases class is the second most represented one and contains a majority of kinases (75% in CSKDE95 and 87% in CS244), which are frequently targeted for anti-cancer therapies as they regulate cell activity. Besides these two predominant protein families, the two benchmark sets contain a large variety of diverse targets.

We additionally assessed the quality and diversity of the complexes and the ligands in the benchmark sets (Figure S2, Tables S3, S4). The resolution and DPI values of the CS405 complexes are globally higher than in CSKDE95, but the selection of 244 complexes out of the CS405 set improves the median resolution and DPI of the structures (Figure S2A and S2B, resp.). The ligand EDIAm values of the CSKDE95 and the CS244 sets are comparable, with 4 (4%) resp. 17 (7%) cases below 0.4, which are potentially problematic as docking references (Figure S2C). The RMSD values between randomized and native ligand structures without (Figure S2D) and with (Figure S2E) superimposition show that our randomization procedure generated notably different ligands geometries, except for small rigid ligands. Randomization of the ligands in CS244 yields higher RMSD values than in CSKDE95, as the former presents ligands with more rotatable dihedrals (Figure S2F). On average, ligands

from CS244 are less buried than ligands from CSKDE95 (Figure S2G). They are more likely to carry a positive charge than a negative charge (Figure S2H), and they are more likely to present crystal contacts than the ligands in CSKDE95 (Figure S2I).

In the SARS-MP-76 cross-docking set, the complexes generally show a good resolution and low DPI, but the ligands display relatively numerous crystal contacts and have a low median EDIAm value (Figure S2A-C,I). Many ligands stem from a fragment-screening study,[2] therefore the median number of rotatable dihedrals is very low (Figure S2F). The cavity of the SARS-CoV-2 main protease is notably open and exposed to solvent (Figure 5), which leads to a median ligand buriedness of only 0.75 (Figure S2G). The cavity contains four sub-cavities[3] (Figure 5), which can be problematic for docking fragments and small ligands, as they can easily bind to different cavities owing to their low specificity. In summary, the SARS-MP-76 set is the most challenging set for docking.

Table S1: Number of complexes and proteins by protein family (c.f. Figure S1).

Benchmark set	CSKDE95		CS244	
Family	#Complexes	#Proteins	#Complexes	#Proteins
Oxidoreductases	4	3	4	3
Transferases	20	9	64	14
Hydrolases	57	34	162	42
Lyases	1	1	0	0
Isomerases	0	0	3	2
Ligases	0	0	1	1
Others	13	10	10	10

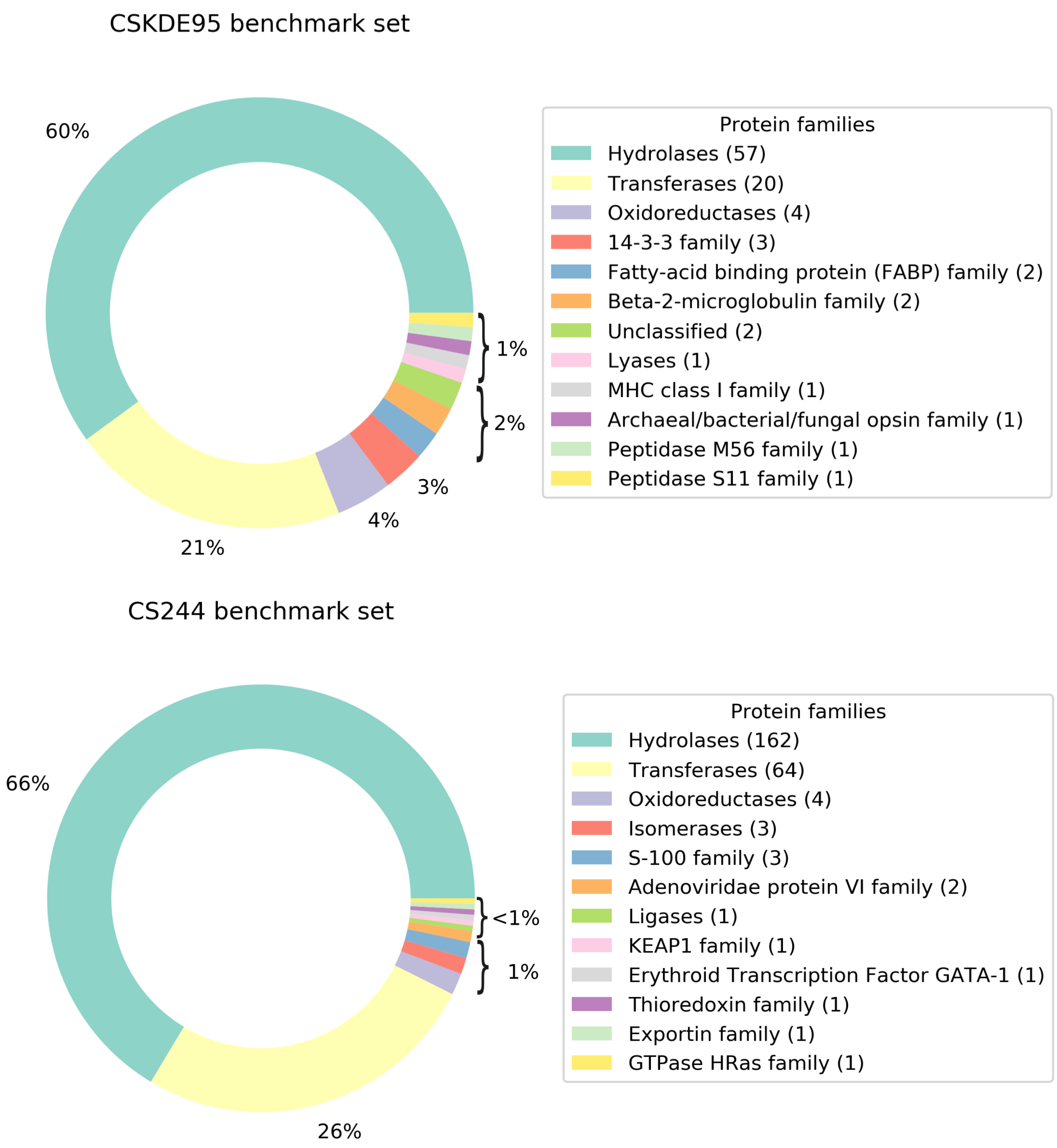

Figure S1: Number of complexes and proteins by protein family.

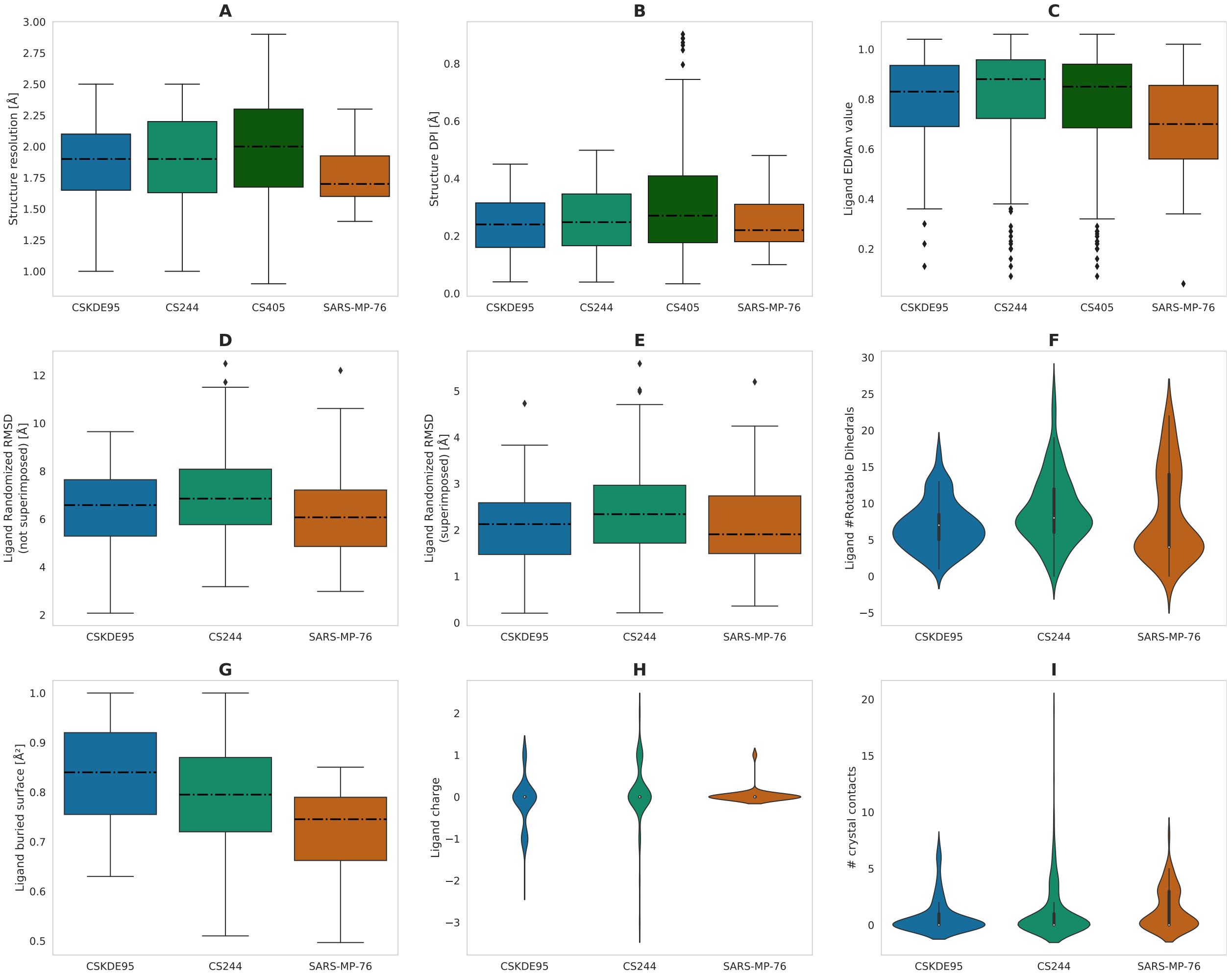

Figure S2: Properties of structures in the CSKDE95 set (blue), the CS244 set (green), the CS405 set (dark green) and the SARS-MP-76 set (orange). A. Resolution of X-ray structure. B. Diffraction-component precision index (DPI). C. Ligand EDIAm values, excluding cases for which it could not be calculated. D. Ligand RMSD between randomized and native pose (not superimposed). E. Ligand RMSD between randomized and native pose (superimposed) F. Number of rotatable ligand dihedrals. G. Portion of buried surface area of the ligand. H. Ligand charge of main configuration at pH 7.4. I. Number of crystal contacts of the ligand.

Analysis of AC Sampling Parameters

Re-docking the CSKDE95 and the CS244 benchmark sets with AC and the *switch* algorithm generally gave particularly high success rates, up to 81% for CSKDE95 and 79% for CS244 when looking at the best pose (RMSD $\leq$ 2 Å, Figure S3, Table S2). The success rate generally increases slightly when using 8 RIC instead of 6. Using a rotational angle of 60° instead of 90° does not improve the results, while the computational time is about four times higher. Therefore, the combination of 8 RIC and a rotational angle of 90° provides a qualitative sampling for a reasonable computational time.

Concerning the concavity value (N_{Thr}), our data shows that all three tested values (50, 60, 70) yield good results for the CSKDE95 set, while for the CS244 set slightly lower success rates are obtained with a value of 70, sampling mainly deep protein cavities. To ensure sufficient sampling with a reasonable computational time for both test sets, we chose to retain a value of $N_{Thr} = 60$ for the rest of this study.

A lower number of rotatable ligand dihedrals and a higher ligand buriedness in the CSKDE95 set (Figure S2F,G) might explain the better success rates when compared to the CS244 set (Figure S3, Table S2). Of note, the top-scored pose of one complex of CSKDE95 has a very high RMSD (11.9 Å). This complex features a highly solvent-exposed ligand, in a small protein devoid of significant cavity and in which crystal contacts were not included in the docking setup (Figure S4C). AC manages to sample a good pose (RMSD $\leq$ 2 Å, Figure S4B), but fails to attribute the best score to it. This example of a scoring failure highlights the influence of structure properties and quality on docking success rates.

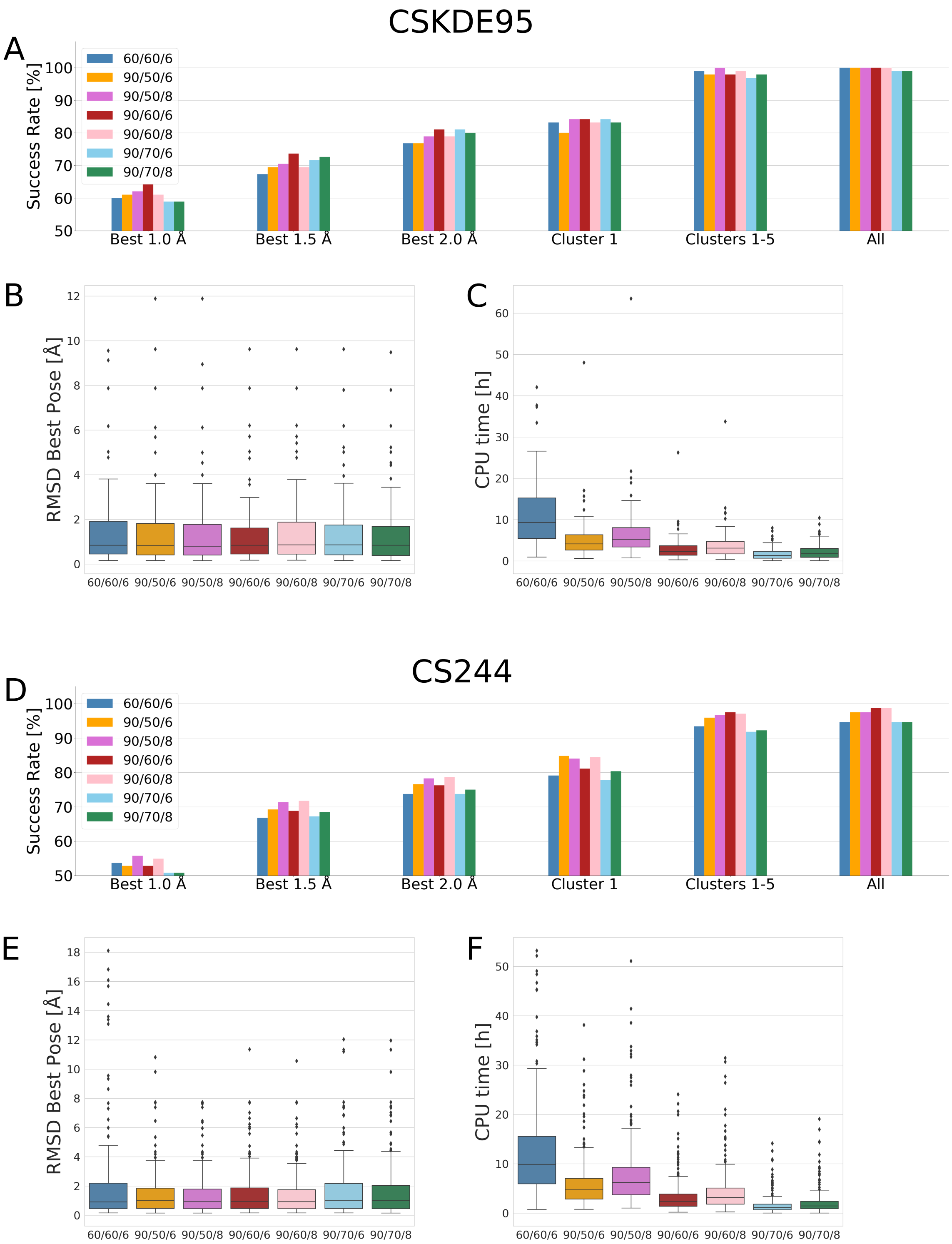

Figure S3: Re-docking results of AC using the *switch* algorithm. Docking conditions are specified as initial rotational angle of the ligand/N_{Thr}/RIC. A/D: Success rates. B/E: RMSD of best pose. C/F: CPU time. Numerical values are given in Table S2.

Table S2: Results from AC re-docking of the CSKDE95 and the CS244 sets with the *switch* method using different sampling parameters (SP: initial ligand rotation [°]/N_{Thr} value/RIC). Median RMSD value of the best pose (RMSD, [Å]); success rate [%] of the best pose RMSD ≤ 1.0 Å (Best-1.0), 1.5 Å (Best-1.5) and 2.0 Å (Best-2.0); success rate [%] of best cluster (Cluster1), best five clusters (Cluster1-5), and in all final poses (All) with an RMSD ≤ 2.0 Å; median CPU time (Time, [h]).

SP	RMSD	Best-1.0	Best-1.5	Best-2.0	Cluster1	Cluster1-5	All	Time
				CSKDE95				
60/60/6	0.84	60	67	77	83	99	100	9.30
90/50/6	0.82	61	69	77	80	98	100	4.14
90/50/8	0.80	62	71	79	84	100	100	5.15
90/60/6	0.84	64	74	81	84	98	100	2.32
90/60/8	0.86	61	69	79	83	99	100	3.09
90/70/6	0.86	59	72	81	84	97	99	1.33
90/70/8	0.84	59	73	80	83	98	99	1.74
				CS244				
60/60/6	0.92	54	67	74	79	93	95	9.89
90/50/6	1.00	53	69	77	85	96	98	4.74
90/50/8	0.93	56	71	78	84	97	98	6.21
90/60/6	0.95	53	69	76	81	98	99	2.40
90/60/8	0.93	55	72	79	84	97	99	3.15
90/70/6	1.02	51	67	74	78	92	95	1.10
90/70/8	1.02	51	68	75	80	92	95	1.48

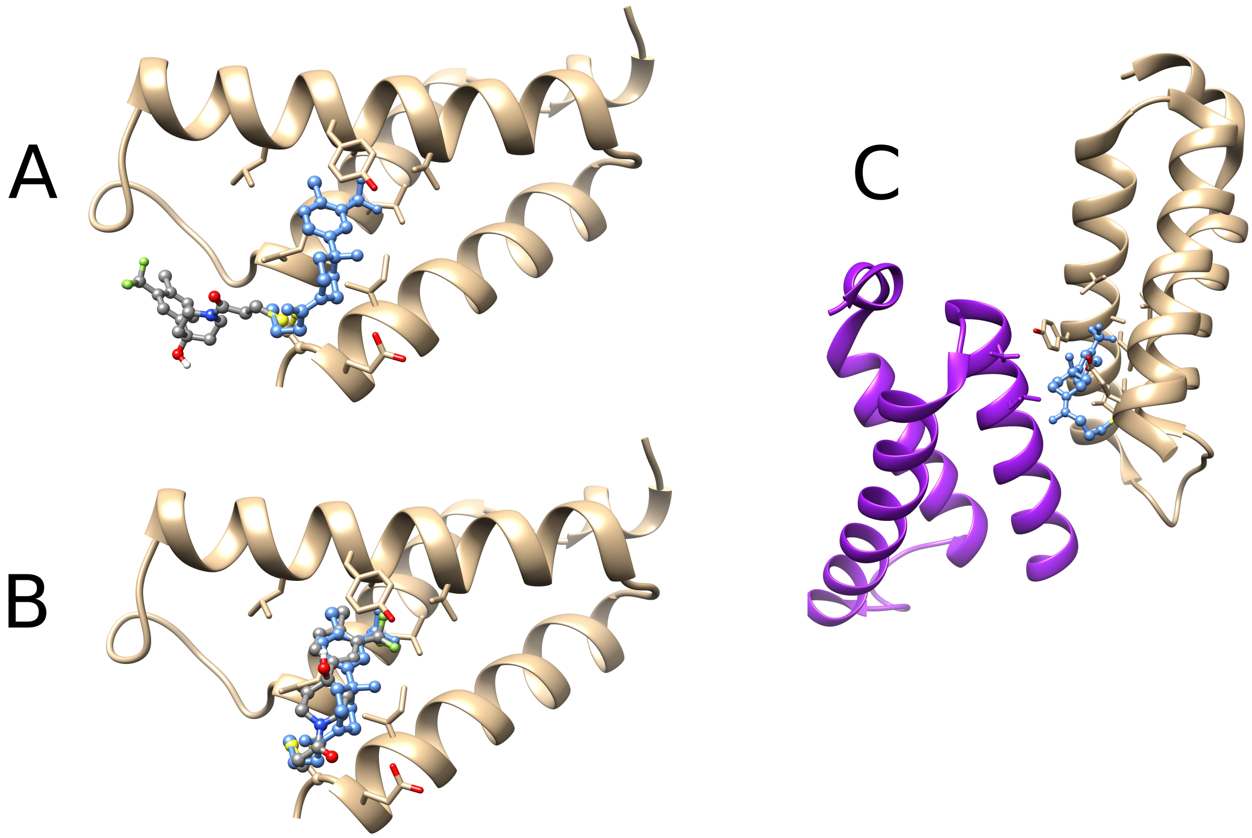

Figure S4: Example of problematic case (PDB ID 4i9o). The ligand (KI1) is represented in ball and stick, the poses from docking are colored by element and the corresponding ligand native pose is colored in blue. A: best pose of the top scored cluster, B: best pose of the second cluster, C: copies of the protein (UniProt ID P45481) involved crystal contacts represented in purple.

Supporting Tables

Table S3: Properties of the CSKDE95 set. Abbreviations used: LIG-ID, ligand identifier from PDB; Rxn, chemical reaction to form covalent bond; Chain, chain identifier from PDB; RMSD, RMSD [Å] between randomized and native ligand conformation after superimposition; CC, number of crystal contacts of the ligand; DOF, number of rotatable dihedrals. Abbreviations for reactions are defined in Figure S5. With additional abbreviations: ald, aldehyde; ket, ketone

PDB-ID	LIG-ID	Rxn	Chain	RMSD	CC	EDIAm	Buriedness	DOF
				CYS				
1nqc	C4P	carbonyle_add(ald)	A	3.45	4	0.77	0.74	13
1td2	PXL	carbonyle_add(ald)	A	0.62	0	0.77	0.97	2
2g8e	0M6	carbonyle_add(ald)	A	2.23	0	0.78	0.75	12
3ovx	O64	carbonyle_add(ald)	A	1.61	0	0.54	0.78	6
3sn8	S89	carbonyle_add(ald)	A	3.71	1	0.82	0.75	12
4cl8	OZJ	carbonyle_add(ald)	A	0.73	0	0.87	0.90	2
5dzj	6B7	carbonyle_add(ald)	A	1.86	0	0.30	0.80	10
5e1i	6B8	carbonyle_add(ald)	A	1.93	0	0.50	0.90	5
6vgy	QZJ	carbonyle_add(ald)	A	3.67	3	-1.00	0.79	13
3h0e	H0E	carbonyle_add(ket)	A	3.13	0	0.68	0.76	5
2awz	5H	michael_add	A	2.27	0	0.74	0.88	5
3svv	VSP	michael_add	A	2.62	0	0.95	0.94	6
3t9t	IAQ	michael_add	A	2.16	0	0.97	0.85	7
4lqm	DJK	michael_add	A	2.61	0	0.74	0.88	5
4qps	37Q	michael_add	A	2.78	0	0.93	0.91	6
5d11	56G	michael_add	A	2.59	0	0.92	0.73	8
5dp4	5E8	michael_add	A	2.36	0	0.36	0.72	11
5j9z	6HJ	michael_add	A	2.29	0	0.88	0.91	4
5p9m	7GB	michael_add	A	1.37	0	-1.00	0.97	6
6iuo	AWX	michael_add	A	1.79	0	-1.00	0.81	6
3kwb	ORH	nitrile_add	X	2.15	0	0.81	0.72	6
3pdf	LXV	nitrile_add	A	2.84	0	0.96	0.87	4
4bs5	MG2	nitrile_add	A	2.25	3	0.94	0.81	7
4cdc	6AO	nitrile_add	B	1.27	0	0.82	0.79	8

4cde	U6B	nitrile_add	B	1.47	0	0.79	0.73	7
4cdf	W2C	nitrile_add	B	1.79	0	0.92	0.75	7
4dmy	0LC	nitrile_add	A	2.58	2	0.91	0.75	5
4x6j	3Y2	nitrile_add	A	2.35	1	0.93	0.79	7
5maj	7KH	nitrile_add	A	3.61	5	0.96	0.70	6
6ezp	C3E	nitrile_add	A	1.76	2	0.91	0.70	3
2dw5	BFB	nucl_subst	A	2.23	0	0.71	0.75	9
2xyg	TQ8	nucl_subst	A	2.15	6	0.13	0.69	7
2xyp	XVE	nucl_subst	A	1.94	7	0.50	0.69	9
5ac0	K9P	nucl_subst	A	2.12	0	0.77	0.84	5
5ac2	K9P	nucl_subst	A	1.47	0	0.74	0.90	5
5l6p	6P8	nucl_subst	A	1.25	0	0.88	0.87	4
5mjb	7O3	nucl_subst	A	1.02	0	0.94	0.88	5
5qh8	H0A	nucl_subst	A	1.36	0	0.70	0.84	3
5qir	J5D	nucl_subst	A	1.48	3	0.75	0.76	3
5rep	T3G	nucl_subst	A	1.50	3	-1.00	0.67	3
1ewl	R99	epoxide_open	A	2.58	2	0.63	0.77	13
1ewm	RL2	epoxide_open	A	1.94	4	0.81	0.74	13
1fj8	CER	epoxide_open	A	2.12	0	0.68	0.97	8
1qdq	074	epoxide_open	A	2.80	0	0.86	0.88	13
2op9	WR1	epoxide_open	B	2.68	0	0.56	0.79	13
3hhi	074	epoxide_open	A	2.81	0	1.00	0.81	13
3qsd	074	epoxide_open	A	2.76	1	0.94	0.88	13
5v6v	8YA	aziridine_open	A	2.06	1	-1.00	0.90	5
6j4p	BJL	epoxide_open	A	2.40	0	-1.00	0.94	10
6j7b	BJL	epoxide_open	A	2.34	0	0.90	0.92	10
3m2z	BFE	disulf_form	A	1.46	0	0.88	0.80	4
3orz	2A2	disulf_form	A	2.27	2	0.90	0.77	5
3otu	J30	disulf_form	A	1.71	4	0.56	0.63	6
4i9o	KI1	disulf_form	A	1.18	2	0.62	0.68	5
4luc	20G	disulf_form	A	2.35	0	0.83	0.94	9
5orl	A4W	disulf_form	A	1.40	0	0.77	0.85	4
5o3y	9JK	disulf_form	A	2.07	2	0.45	0.67	3

5vbm	92V	disulf_form	A	1.18	6	1.00	0.71	8
6hmu	GE8	disulf_form	A	1.88	0	0.64	0.81	7
6hn2	GF8	disulf_form	A	1.63	0	0.59	0.76	5
SER								
2q9m	LK7	blac_open	A	1.99	1	0.65	0.79	4
3mzd	CXV	blac_open	A	2.90	0	0.65	0.84	7
3q7z	BOU	blac_open	A	3.09	0	0.97	0.74	8
3zmh	L62	blac_open	A	1.50	0	0.94	1.00	7
4mll	1S6	blac_open	A	2.26	0	0.65	0.92	7
5ty2	NFF	blac_open	A	2.76	1	-1.00	0.87	8
5xhr	PXH	blac_open	A	2.89	0	0.77	0.87	7
4jxg	1S6	blac_open	A	1.38	0	0.83	0.85	7
1ghm	CED	blac_open	A	1.48	1	0.84	0.85	7
1hv7	616	glac_open	A	1.45	2	0.96	0.72	9
1xrl	PHK	carbonyle_add(ket)	A	0.75	0	-	1.00	4
1zpb	995	carbonyle_add(ket)	A	3.83	1	0.94	0.79	16
3k7f	F2C	carbonyle_add(ket)	A	2.50	0	0.98	0.98	10
1hbj	FBQ	carbonyle_add(ket)	A	3.20	0	0.90	0.99	6
2wj1	S99	carbonyle_add(ket)	A	2.84	0	0.97	0.98	9
3oj8	OJ8	carbonyle_add(ket)	A	2.44	0	1.01	0.98	5
4amy	2P2	carbonyle_add(ket)	A	3.21	0	0.22	0.80	10
4amz	2P4	carbonyle_add(ket)	A	2.42	0	0.99	0.84	8
4an1	2P8	carbonyle_add(ket)	A	1.81	0	0.96	0.83	8
6ary	BT7	carbonyle_add(ket)	A	1.12	0	0.71	0.99	5
LYS								
2z9w	PXL	imine_form	A	0.82	0	0.94	0.98	2
4qgv	LMC	imine_form	A	1.06	0	0.90	0.95	3
5u17	7WP	imine_form	A	0.20	0	-1.00	1.00	1
6aff	72U	imine_form	A	1.07	0	0.94	0.66	2
6c7z	JLO	imine_form	A	1.24	0	0.44	0.98	3
6pud	OYD	imine_form	A	1.83	0	-1.00	1.00	8
6puh	OYG	imine_form	A	0.99	0	1.04	0.99	2

6eej	J6S	imine_form	A	0.79	0	0.88	0.98	5
6wp8	RET	imine_form	A	2.36	0	-1.00	1.00	5
6yq2	P7T	imine_form	A	0.58	0	-1.00	0.88	3
ASP								
4wsk	3U2	epoxide_open	A	1.96	0	0.85	0.79	3
5vnp	9FM	nucl_subst	A	4.73	6	0.65	0.74	17
5uxz	8PM	nucl_subst	A	2.74	1	0.90	0.95	11
GLU								
6q6l	RJR	epoxide_open	A	2.54	0	0.71	0.95	8
5d6e	94A	epoxide_open	A	1.78	0	0.89	0.86	7

Table S4: Properties of the CS244 set. Abbreviations used: LIG-ID, ligand identifier from PDB, Asterisk: ligand composed of several fragments, 3-letter code given for the binding fragment; Rxn, chemical reaction to form covalent bond; Chain, chain identifier from PDB where the ligand is placed; RMSD, RMSD [Å] between randomized and native ligand conformation after superimposition; CC, number of crystal contacts of the ligand; DOF, number of rotatable dihedrals. Abbreviations for reactions are defined in Figure S5. With additional abbreviations: ald, aldehyde; ket, ketone

PDB-ID	LIG-ID	Rxn	Chain	RMSD	CC	EDIAm	Buriedness	DOF
CYS								
1ewl	R99	epoxide_open	A	2.58	2	0.63	0.77	13
1ewm	RL2	epoxide_open	A	1.94	4	0.81	0.74	13
1gmy	AEM*	nitrile_add	A	2.70	0	0.97	0.93	10
1hvy	UMP	michael_add	A	1.22	0	-1.00	1.00	4
1m6d	MYP	michael_add	A	3.38	6	0.98	0.62	14
1nfz	EIP	epoxide_open	A	1.87	0	0.68	0.98	7
1nms	161	nucl_subst	A	2.08	1	1.01	0.81	11
1npz	C1P	michael_add	A	4.28	2	0.97	0.71	15
1nqc	C4P	carbonyle_add(ald)	A	3.45	4	0.77	0.74	13
1pyo	ASJ	carbonyle_add(ald)	A	4.29	0	0.79	0.76	3
1rhj	PZN	carbonyle_add(ket)	A	3.96	0	-1.00	0.76	19
1rwn	4QB	carbonyle_add(ket)	A	2.63	0	0.88	0.74	16
1rwp	HQC	carbonyle_add(ket)	A	3.62	0	0.80	0.69	14
1rwx	YBH	carbonyle_add(ket)	A	2.68	3	0.50	0.72	16
1u9v	IHE	nitrile_add	A	1.04	6	0.94	0.61	5
2a5k	AZP	epoxide_open	A	4.37	0	0.72	0.87	24
2alv	CY6	michael_add	A	3.34	0	0.36	0.81	18
2amd	9IN	michael_add	A	3.74	0	0.72	0.81	22
2aux	CT1	carbonyle_add(ket)	A	1.83	4	0.78	0.74	11
2auz	CT2	carbonyle_add(ket)	A	2.43	1	0.74	0.70	10
2awz	5H	michael_add	A	2.27	0	0.74	0.88	5
2ax0	5X	michael_add	A	2.19	0	0.84	0.87	6
2ax1	5EE	michael_add	A	3.40	0	0.92	0.87	5
2bdl	4PR	carbonyle_add(ket)	A	4.39	4	0.76	0.62	15
2c2k	AA1	michael_add	A	5.02	0	0.69	0.72	8

2cno	M60	epoxide_open	A	5.59	0	0.27	0.71	25
2dcc	77B	epoxide_open	A	4.16	10	0.91	0.60	15
2dw5	BFB	nucl_subst	A	2.23	0	0.71	0.75	9
2f7d	NOQ	nitrile_add	A	2.01	3	0.92	0.76	8
2fq9	CRJ	nitrile_add	A	3.66	0	0.97	0.81	11
2fra	CRV	nitrile_add	A	2.53	0	0.98	0.81	12
2frq	C71	nitrile_add	A	3.16	0	0.89	0.69	10
2ft2	C28	nitrile_add	A	2.68	0	1.00	0.80	11
2fud	CRL	nitrile_add	A	1.74	0	0.88	0.76	8
2fye	BCQ	michael_add	A	4.42	3	0.58	0.71	17
2g6d	MQQ	michael_add	A	3.57	3	0.76	0.69	17
2g8e	0M6	carbonyle_add(ket)	A	2.23	0	0.78	0.75	12
2gh5	ELI	nucl_subst	A	2.44	0	0.62	0.88	6
2gke	ZDP	aziridine_open	A	1.89	0	1.00	1.00	6
2gkj	ZDR	aziridine_open	A	2.15	0	0.99	1.00	6
2gx4	NOL	carbonyle_add(ket)	A	3.91	1	0.93	0.79	19
2hwo	RBS	michael_add	A	1.24	0	0.88	0.87	5
2hwp	DJK	michael_add	A	1.52	0	0.88	0.84	5
2op9	WR1	epoxide_open	B	2.68	0	0.56	0.79	13
2qlq	SR2	michael_add	B	2.76	0	0.47	0.81	7
2qq7	SR2	michael_add	A	2.46	0	0.77	0.88	7
2r6n	CKE	nitrile_add	A	1.89	3	0.97	0.68	5
2r9f	K2Z	carbonyle_add(ket)	A	3.76	1	0.70	0.59	18
2xm7	34H	carbonyle_add(ket)	A	1.11	0	0.89	0.99	3
2xu1	424	nitrile_add	A	3.04	0	0.95	0.72	9
2xu4	DJT	nitrile_add	A	3.04	5	1.03	0.70	9
2xu5	XU5	nitrile_add	A	2.47	4	1.03	0.71	9
2xyg	TQ8	nucl_subst	A	2.15	6	0.13	0.69	7
2xyp	XVE	nucl_subst	A	1.94	7	0.50	0.69	9
2yj2	YJ2	nitrile_add	A	3.02	4	1.00	0.73	9
2yj8	YJ8	nitrile_add	A	1.87	4	0.97	0.73	9
2yj9	YJ9	nitrile_add	A	2.86	2	1.01	0.72	10
2yjb	YJ9	nitrile_add	A	2.79	6	1.01	0.72	10

2yjc	424	nitrile_add	A	3.19	3	1.01	0.72	9
2zk2	PTG	michael_add	A	2.67	0	0.51	0.99	11
2zu5	ZU5	michael_add	A	4.29	1	0.98	0.80	22
3b1u	YFF	nucl_subst	A	1.73	0	0.86	0.78	11
3blu	PVS	michael_add	A	1.55	0	0.23	0.96	2
3bwk	C1P	michael_add	C	3.93	0	0.85	0.73	15
3h0e	H0E	carbonyle_add(ket)	A	3.13	0	0.68	0.76	5
3hd3	25B	michael_add	A	2.38	0	0.89	0.69	12
3hha	NOW	nitrile_add	A	2.60	0	0.93	0.73	10
3i06	QL2	nitrile_add	A	0.90	0	0.86	0.71	3
3kjn	B93	nucl_subst	A	4.27	0	0.97	0.73	13
3kw9	ORG	nitrile_add	A	1.81	1	0.87	0.70	3
3kwb	ORH	nitrile_add	X	2.15	0	0.81	0.72	6
3kwz	KWZ	nitrile_add	A	2.09	3	0.98	0.66	6
3lok	DJK	michael_add	A	1.44	0	0.71	0.87	5
3o1g	O75	nitrile_add	A	2.36	4	0.83	0.62	11
3o6t	PX5	michael_add	A	0.80	0	0.91	0.73	1
3of8	I0Y	carbonyle_add(ket)	A	2.44	6	0.29	0.70	16
3of9	I0X	nucl_subst	A	2.58	7	0.99	0.72	16
3orz	2A2	disulf_form	A	2.27	2	0.90	0.77	5
3otu	J30	disulf_form	A	2.15	4	0.56	0.63	6
3ovx	O64	carbonyle_add(ket)	A	1.61	0	0.54	0.78	6
3rhy	HM3	nucl_subst	A	0.46	0	0.38	0.97	1
3s3q	C1P	michael_add	A	3.92	1	0.96	0.77	15
3sn8	S89	carbonyle_add(ald)	A	3.71	1	0.82	0.75	12
3svv	VSP	michael_add	A	2.62	0	0.95	0.94	6
3t9t	IAQ	michael_add	A	2.16	0	0.97	0.85	7
3v4j	PQR	nucl_subst	A	0.56	0	0.78	0.83	2
3w2p	W2P	michael_add	A	3.40	0	0.93	0.78	13
3w2q	HKI	michael_add	A	3.29	0	0.86	0.78	13
3zv9	G74	michael_add	A	1.55	0	0.80	0.70	11
3zvg	G86	michael_add	A	3.42	0	0.79	0.66	22
4bpv	OFH	nitrile_add	C	3.41	0	0.97	0.93	12

4bqv	8PW	nitrile_add	A	3.33	0	0.98	0.84	12
4bs5	MG2	nitrile_add	A	2.25	3	0.94	0.81	7
4bs6	JG7	nitrile_add	A	2.94	3	0.95	0.65	11
4bsq	QQV	nitrile_add	A	2.54	1	0.86	0.76	8
4cdc	6AO	nitrile_add	B	1.27	0	0.82	0.79	8
4cdd	GDI	nitrile_add	B	2.58	0	0.87	0.77	7
4cde	U6B	nitrile_add	B	1.47	0	0.79	0.73	7
4cdf	W2C	nitrile_add	B	1.79	0	0.92	0.75	7
4d9u	0JH	michael_add	A	1.40	0	0.61	0.87	9
4dcd	K36	carbonyle_add(ket)	A	2.49	0	0.98	0.78	14
4dmx	0LB	nitrile_add	A	1.53	4	0.86	0.71	6
4dmy	0LC	nitrile_add	A	2.58	2	0.91	0.75	5
4gmx	K85	michael_add	C	2.14	0	0.73	0.91	8
4gs6	1FM	michael_add	A	0.69	0	0.80	0.92	1
4hcu	13L	michael_add	A	1.41	0	1.01	0.89	8
4hcv	13J	michael_add	A	2.08	0	1.00	0.89	8
4lqm	DJK	michael_add	A	2.61	0	0.74	0.88	5
4luc	20G	disulf_form	A	2.35	0	0.83	0.94	9
4lv6	20H	disulf_form	A	2.45	0	1.00	0.95	8
4lyf	21C	michael_add	B	1.92	0	1.00	0.82	7
4lyh	21F	michael_add	B	2.56	0	0.99	0.83	8
4lyj	21F	michael_add	A	1.91	0	0.94	0.83	8
4m1o	21J	michael_add	B	2.33	0	0.89	0.84	7
4m1s	21K	michael_add	B	2.09	0	0.95	0.81	7
4m1t	21M	michael_add	B	2.33	0	0.81	0.82	7
4m1w	21R	michael_add	A	2.39	0	0.41	0.82	8
4m1y	21S	michael_add	B	2.22	0	0.96	0.82	7
4m22	22C	michael_add	B	1.36	4	0.97	0.85	6
4mzo	2EW	nitrile_add	A	1.84	0	0.93	0.78	8
4mzs	2EV	nitrile_add	A	2.10	0	0.85	0.87	8
4pdz	CTI	michael_add	B	0.72	2	0.87	0.63	2
4pe0	NQS	nucl_subst	A	0.78	4	0.40	0.68	3
4pe7	ODN	michael_add	A	0.31	2	0.88	0.51	0

4pi3	2V5	michael_add	A	4.45	0	0.94	0.83	18
4piq	3FS	nitrile_add	A	2.08	0	0.65	0.86	12
4pis	3FU	nitrile_add	A	3.20	1	0.69	0.84	12
4qps	37Q	michael_add	A	2.78	0	0.93	0.91	6
4qq5	37O	michael_add	A	2.38	0	0.87	0.81	11
4qqc	37O	michael_add	A	3.39	0	0.75	0.81	11
4thi	PYD	nucl_subst	A	0.21	0	0.89	0.92	0
4twy	3BL	carbonyle_add(ket)	A	2.90	1	0.90	0.81	9
4us2	L7S	michael_add	R	1.45	0	0.27	0.72	3
4wx4	3VF	nitrile_add	A	2.32	0	0.93	0.86	8
4x0t	3W9	carbonyle_add(ket)	A	0.88	0	0.71	0.96	4
4x6j	3Y2	nitrile_add	A	2.35	1	0.93	0.79	7
4xcu	40M	michael_add	A	2.01	0	0.99	0.86	8
4xz0	4N5	nucl_subst	A	1.42	8	0.62	0.61	5
4yhf	4C9	michael_add	A	1.41	0	0.89	0.93	8
4yqm	4G9	nucl_subst	A	1.97	0	0.67	0.90	5
4yrt	781	michael_add	A	1.20	0	0.98	0.89	3
4yv8	LIG	carbonyle_add(ket)	A	3.37	0	-1.00	0.59	23
4zzm	CQ6	michael_add	A	1.60	0	0.98	0.87	4
4zzo	CQ3	michael_add	A	2.84	0	1.06	0.91	7
5c1u	GHV	carbonyle_add(ket)	A	2.88	1	-1.00	0.65	13
5c1x	GHX	carbonyle_add(ket)	A	1.42	0	-1.00	0.76	13
5c1y	GHY	carbonyle_add(ket)	A	3.22	0	0.20	0.73	12
5c5o	SDJ	carbonyle_add(ket)	A	2.37	0	0.88	0.82	11
5d11	56G	michael_add	A	2.59	0	0.92	0.73	8
5daf	58E	michael_add	A	0.74	0	0.63	0.73	1
5dp4	5E8	michael_add	A	2.36	0	0.36	0.72	11
5dp5	5E9	michael_add	A	2.88	0	0.75	0.71	15
5dp7	5EC	michael_add	A	3.13	0	0.56	0.71	15
5dp9	5EX	michael_add	A	4.98	0	0.60	0.74	17
5dpa	5F2	michael_add	A	2.23	0	0.75	0.75	13
5e7r	5KW	nucl_subst	A	2.48	0	0.95	0.76	8
5e93	5LL	michael_add	A	0.65	0	-1.00	1.00	2

5ea9	5LM	michael_add	A	1.23	0	-1.00	1.00	2
5f02	5T9	nitrile_add	A	3.21	5	1.02	0.69	10
5f2e	5UT	michael_add	A	3.06	1	0.98	0.98	8
5fx5	HOV	michael_add	A	3.46	2	0.97	0.75	17
5fx6	6OY	michael_add	A	3.20	5	0.68	0.66	17
5gnk	80U	michael_add	A	2.75	0	1.01	0.93	7
5hg5	633	michael_add	A	4.71	2	0.75	0.73	8
5hg7	630	michael_add	A	2.61	0	0.92	0.83	8
5hg8	634	michael_add	A	3.40	1	1.01	0.81	7
5hg9	63A	michael_add	A	2.59	0	0.78	0.83	8
5hze	E62	michael_add	A	0.66	0	0.99	0.96	1
5iyt	NZN	michael_add	B	1.48	4	0.25	0.56	8
5j87	N42	michael_add	A	3.79	0	0.97	0.78	12
5j9z	6HJ	michael_add	A	2.29	0	0.88	0.91	4
5l6o	6P6	nucl_subst	A	0.66	0	0.89	0.97	3
5l6p	6P8	nucl_subst	A	1.25	0	0.88	0.87	4
5lcj	6TS	michael_add	A	4.64	2	0.22	0.58	12
5lck	6TT	michael_add	A	1.93	0	0.91	0.91	9
5maj	7KH	nitrile_add	A	3.61	5	0.96	0.70	6
5mjb	7O3	nucl_subst	A	1.02	0	0.94	0.88	5
5mqy	GH4	nitrile_add	A	2.31	0	0.94	0.76	8
5n0y	8FQ	nucl_subst	A	2.46	0	0.93	0.76	11
5n19	D03	carbonyle_add(ket)	A	4.03	0	0.65	0.76	16
5o8u	9O5	michael_add	A	2.65	1	0.95	0.93	8
5o8v	9O2	michael_add	A	1.56	1	0.76	0.88	8
5p9j	8E8	michael_add	A	1.36	0	1.05	0.89	6
5p9k	7G8	michael_add	A	3.36	0	1.06	0.65	11
5p9m	7GB	michael_add	A	1.37	0	-1.00	0.97	6
5tdi	7AS	nitrile_add	A	1.50	3	-1.00	0.68	12
5tg2	V69	carbonyle_add(ket)	A	1.52	1	-1.00	0.70	7
5th7	7BY	michael_add	A	1.45	0	1.01	0.98	10
5toz	7H4	michael_add	A	1.24	0	0.95	0.87	4
5tts	7KU	michael_add	A	1.11	0	0.96	0.88	4

5ttu	7KV	michael_add	A	2.15	0	0.99	0.92	3
5ttv	7KX	michael_add	A	2.30	0	1.01	0.88	4
5ug8	8BP	michael_add	A	2.03	0	1.02	0.84	7
5ug9	8AM	michael_add	A	2.53	0	1.03	0.85	8
5ugc	8BS	michael_add	A	1.28	0	1.00	0.84	7
5v6v	8YA	aziridine_open	A	2.06	1	-1.00	0.90	5
5v71	8ZG	michael_add	A	1.65	0	0.91	0.80	4
5v88	8ZD	michael_add	A	3.73	2	-1.00	0.84	13
5v9o	91G	michael_add	A	1.43	0	0.69	0.86	9
5vnd	9ES	michael_add	A	2.37	0	0.76	0.74	13
5vqx	9HY	nucl_subst	A	2.05	0	-1.00	0.98	8
5vqy	9J1	michael_add	A	2.71	0	-1.00	0.98	9
5wfj	9Z4	michael_add	A	2.13	0	0.90	0.90	7
6ay2	C1G	nitrile_add	A	2.58	2	0.16	0.64	13
6ffn	D8K	carbonyle_add(ket)	A	1.07	0	0.88	0.76	6
			SER					
1haz	IL0*	carbonyle_add(ket)	B	2.22	0	0.20	0.76	15
1zsl	624	carbonyle_add(ket)	A	3.08	4	0.87	0.81	14
2ex6	AIX	blac_open	A	2.30	0	0.99	0.68	7
2ex8	PNM	blac_open	A	2.22	7	0.96	0.70	7
2ex9	35P	blac_open	A	2.60	0	0.94	0.70	8
2exb	FXM	blac_open	A	3.89	13	0.50	0.69	16
2h5s	SA2	blac_open	A	3.25	0	0.76	0.75	13
2i03	AXD	nitrile_add	B	2.88	0	0.90	0.73	7
2j8y	PNM	blac_open	A	2.67	9	0.69	0.87	7
2jbf	PNM	blac_open	A	2.33	0	0.09	0.87	7
2oc0	HU1	carbonyle_add(ket)	A	3.42	0	0.85	0.66	14
2px6	DH9	carbonyle_add(ald)	A	2.55	0	0.48	0.78	26
2v35	J54	carbonyle_add(ket)	A	2.10	4	0.63	0.80	7
2vgj	REC	blac_open	A	2.60	0	0.93	0.81	11
2wke	BIY	carbonyle_add(ket)	A	0.82	0	0.74	0.75	2
3a3e	CMV	blac_open	B	2.71	0	0.84	0.55	10
3a3f	FMZ	blac_open	B	2.57	0	0.89	0.82	9

3a3i	AIX	blac_open	B	2.42	0	1.02	0.74	7
3bfc	IM2	blac_open	A	1.78	1	0.60	0.81	9
3bff	SFR	blac_open	A	1.31	0	0.70	0.87	5
3bfg	MER	blac_open	A	3.03	5	0.20	0.74	8
3bjm	BJM	nitrile_add	A	2.10	0	0.95	0.85	4
3cg5	ISS	blac_open	A	1.52	1	0.85	0.83	6
3k84	K84	carbonyle_add(ket)	A	3.34	0	0.79	0.99	17
3lj6	PIX	carbonyle_add(ket)	A	1.99	0	0.93	0.99	6
3mzd	CXV	blac_open	A	2.90	0	0.65	0.84	7
3pae	4J6	blac_open	A	2.67	0	0.92	0.83	9
3upn	IM2	blac_open	B	1.62	0	0.53	0.95	9
3upo	PNM	blac_open	B	2.01	0	0.38	0.92	7
3upp	CEF	blac_open	B	1.68	0	0.67	0.91	8
3w2t	LF7	nitrile_add	A	1.02	0	0.98	0.86	5
3zfz	AI8	blac_open	A	3.16	19	0.75	0.83	12
4ben	IM2	blac_open	A	1.46	0	0.35	0.70	9
4q8i	TEB	blac_open	A	2.04	0	0.84	0.72	7
4uuq	64D	carbonyle_add(ket)	A	2.12	0	0.84	0.93	5
4ym9	4E4	carbonyle_add(ket)	A	0.84	0	0.83	0.80	6
5ced	PNM	blac_open	A	1.60	0	0.90	0.82	7
5eph	ID1	blac_open	A	1.93	2	0.76	0.74	8
5kre	6WG	carbonyle_add(ket)	A	1.03	0	0.94	0.85	2

Table S5: Properties of the SARS-MP-76 set. Abbreviations used: LIG-ID, ligand identifier from PDB. asterisk: ligand composed of several fragments, 3-letter code given for the binding fragment; Rxn, chemical reaction to form covalent bond; Chain, chain identifier from PDB where the ligand is placed; RMSD, RMSD [Å] between randomized and native ligand conformation after superimposition; CC, number of crystal contacts of the ligand; EDIAm values; Confidence in structure from Ref.,[2] H: high, L: low, hyphen: not provided. DOF, number of rotatable dihedrals. Abbreviations for reactions are defined in Figure S5. With additional abbreviations: ald, aldehyde; ket, ketone

PDB-ID	LIG-ID	Rxn	Chain	RMSD	CC	EDIAm	Confidence	Buriedness	DOF
5rej	T1V	nucl_subst	A	1.51	2	-1	H	0.57	4
5rek	T1Y	nucl_subst	A	1.19	3	0.63	H	0.56	4
5rel	T2G	nucl_subst	A	1.66	0	-1	L	0.79	4
5rem	T2J	nucl_subst	A	1.25	0	0.58	L	0.77	4
5ren	T2V	nucl_subst	A	1.81	0	-1	H	0.85	3
5reo	T2Y	nucl_subst	A	1.91	0	0.37	L	0.64	4
5rep	T3G	nucl_subst	A	2.32	5	-1	H	0.56	4
5rer	T3J	nucl_subst	A	1.55	0	-1	H	0.84	3
5res	T3V	nucl_subst	A	1.80	3	-1	H	0.55	4
5ret	T47	nucl_subst	A	1.44	0	-1	L	0.83	4
5reu	T4D	nucl_subst	A	2.11	0	0.59	H	0.79	4
5rev	T4J	nucl_subst	A	1.92	0	-1	L	0.62	5
5rew	T4M	nucl_subst	A	1.27	0	-1	L	0.74	4
5rex	T4V	nucl_subst	A	1.20	1	-1	L	0.77	4
5rey	T4Y	nucl_subst	A	2.25	2	-1	H	0.84	4
5rff	T6M	nucl_subst	A	2.37	3	-1	H	0.58	4
5rfh	T6Y	nucl_subst	A	1.86	0	-1	L	0.76	4
5rfi	T71	nucl_subst	A	2.68	0	-1	H	0.78	4
5rfj	T7A	nucl_subst	A	1.50	0	-1	H	0.77	4
5rfk	T7D	nucl_subst	A	1.52	3	-1	L	0.62	5
5rfl	T7G	nucl_subst	A	2.13	3	-1	L	0.60	5
5rfm	T7J	nucl_subst	A	1.77	0	0.56	L	0.76	4
5rfn	T7P	nucl_subst	A	0.69	0	-1	H	0.74	4
5rfo	T7S	nucl_subst	A	0.65	0	0.49	L	0.75	4
5rfp	T7V	nucl_subst	A	1.44	0	-1	H	0.83	4

5rfq	T7Y	nucl_subst	A	1.56	3	-1	H	0.54	4
5rfr	T81	nucl_subst	A	0.92	0	-1	L	0.80	4
5rfs	T84	nucl_subst	A	1.90	0	-1	H	0.84	4
5rft	T8A	nucl_subst	A	0.47	0	-1	H	0.84	3
5rfu	T8D	nucl_subst	A	2.27	2	-1	H	0.56	4
5rfv	T8J	nucl_subst	A	1.67	0	0.56	L	0.74	4
5rfw	T8M	nucl_subst	A	1.27	0	-1	H	0.79	4
5rfx	T8P	nucl_subst	A	1.47	4	-1	L	0.51	4
5rfy	T8S	nucl_subst	A	1.81	0	-1	H	0.77	5
5rfz	T8V	nucl_subst	A	0.89	0	-1	H	0.74	3
5rg0	T8Y	nucl_subst	A	1.35	0	-1	H	0.75	3
5rgl	U0Y	nucl_subst	A	1.51	3	-1	L	0.54	4
5rgm	U1D	nucl_subst	A	1.93	0	-1	L	0.82	5
5rgn	U1A	nucl_subst	A	1.73	2	-1	L	0.56	4
5rgo	U1G	nucl_subst	A	2.13	1	-1	L	0.75	4
5rgp	U1M	nucl_subst	A	1.45	3	-1	L	0.57	4
5rgt	UHS	michael_add	A	2.92	3	0.06	-	0.81	9
5rh5	UHV	michael_add	A	2.45	5	0.34	-	0.79	10
5rh6	UHY	michael_add	A	2.98	4	0.56	-	0.74	10
5rh7	UJ1	michael_add	A	2.15	4	0.53	-	0.79	10
5rh9	UJ4	michael_add	A	2.61	8	0.59	-	0.78	10
5rha	T8M	nucl_subst	A	1.56	0	-1	-	0.80	4
5rhb	USD	nitrile_add	A	0.36	0	-1	H	0.69	0
5rhe	UPD	nucl_subst	A	1.05	5	0.36	L	0.53	6
5rhf	UPJ	nucl_subst	A	1.21	1	-1	H	0.73	5
6lu7	PJE*	michael_add	C	4.24	1	0.95	-	0.71	22
6lze	FHR	carbonyle_add(ald)	A	3.24	1	0.94	-	0.73	11
6m0k	FJC	carbonyle_add(ald)	A	2.00	1	0.7	-	0.71	11
6wnp	U5G	carbonyle_add(ket)	A	1.80	0	0.95	-	0.81	14
6wtj	K36	carbonyle_add(ald)	A	3.53	3	-1	-	0.76	14
6wtk	UED	carbonyle_add(ald)	A	2.58	4	-1	-	0.50	14
6wtt	K36	carbonyle_add(ald)	A	1.87	0	-1	-	0.74	14
6xa4	UXS*	carbonyle_add(ald)	B	2.79	0	0.59	-	0.77	16

6xbg	UZ4∗	carbonyle_add(ket)	A	3.12	0	-1	-	0.76	16
6xbh	ELL∗	carbonyle_add(ald)	A	2.86	0	-1	-	0.74	16
6xbi	UZ4∗	carbonyle_add(ket)	D	2.31	0	-1	-	0.75	16
6xfn	V1V∗	carbonyle_add(ket)	B	4.05	0	-1	-	0.59	16
6xhm	V2M	carbonyle_add(ket)	A	3.28	0	1.02	-	0.75	14
6xmk	QYS	carbonyle_add(ald)	A	1.67	0	0.86	-	0.74	19
6xqs	SV6	carbonyle_add(ket)	A	3.18	0	0.92	-	0.73	20
6xqt	NNA	carbonyle_add(ket)	A	5.20	4	0.73	-	0.78	13
6xqu	U5G	carbonyle_add(ket)	A	2.48	3	0.73	-	0.82	1
6xr3	V7G	carbonyle_add(ket)	A	3.41	1	0.85	-	0.75	22
6ynq	P6N	michael_add	A	0.36	3	0.86	-	0.74	19
6zrt	SV6	carbonyle_add(ket)	A	3.44	5	0.82	-	0.67	22
6zru	U5G	carbonyle_add(ket)	A	3.51	3	-1	-	0.82	19
7bqy	PJE∗	michael_add	C	3.33	1	1.00	-	0.53	22
7c7p	SV6	carbonyle_add(ket)	A	4.01	0	0.76	-	0.73	19
7c8r	TG3	aziridine_open	A	4.20	1	0.69	-	0.77	14
7c8t	NOL	carbonyle_add(ald)	A	3.88	1	0.78	-	0.68	14
7com	U5G	carbonyle_add(ket)	A	2.72	0	0.77	-	0.81	13

Table S6: Results from AC re-docking for CSKDE304 with the *non-cov*, *cov-only* and *switch* methods. The *switch* method was additonally used with two different cutoffs for covalent docking: 5 Å and 10 Å (cutoff5 and cutoff10, resp.) SP are 90°/N_{Thr}=60/8 RIC; median RMSD value of the best pose (RMSD, [Å]); success rate [%] of the best pose RMSD $\leq$ 1.0 Å (Best-1.0), 1.5 Å (Best-1.5) and 2.0 Å (Best-2.0); success rate [%] of best cluster (Cluster1), best five clusters (Cluster1-5), and in all final poses (All) with an RMSD $\leq$ 2.0 Å; median CPU time (Time, [h]).

AC procedure	RMSD	Best-1.0	Best-1.5	Best-2.0	Cluster1	Cluster1-5	All	Time
				CSKDE304				
non-cov	3.23	24	40	51	56	73	87	3.78
cov-only	1.57	52	68	75	81	96	98	4.02
cutoff5	1.05	50	64	72	78	93	96	2.99
cutoff10	0.93	55	70	77	82	95	98	3.77
switch	1.43	57	71	78	83	97	99	4.51

Table S7: Comparison of re-docking results obtained with AC, AD and GOLD on the CSKDE304 and CSKDE212 sets. Docking from the randomized (R) or native (N) ligand conformation. AC with *non-cov* (NC), *cov-only* (CO) and *switch* (S); GOLD with Chemscore (CS), Goldscore (GS) and PLP scoring functions; GOLD and AD were run additionally with 1000 GA, to get a long (L) docking. Median RMSD value of the best pose (RMSD, [Å]); success rate [%] of the best pose RMSD ≤ 1.0 Å (Best-1.0), 1.5 Å (Best-1.5) and 2.0 Å (Best-2.0); success rate [%] of best cluster (Cluster1), best five clusters (Cluster1-5), and in all final poses (All) with an RMSD ≤ 2.0 Å; median CPU time (Time, [h]).

Docking	RMSD	Best-1.0	Best-1.5	Best-2.0	Cluster1	Cluster1-5	All	Time
				**CSKDE304**				
				AC				
NC/R	3.23	24	40	51	56	73	87	3.8
CO/R	1.57	52	68	75	81	96	98	4.0
S/R	1.43	57	71	78	83	97	99	4.5
S/N	1.26	62	76	83	84	98	99	4.7
				GOLD				
CS/R	2.30	29	49	63	70	85	85	0.03
GS/R	2.31	30	49	62	70	89	90	0.19
PLP/R	2.18	34	55	66	74	89	89	0.03
PLP/R-L	2.10	35	56	68	78	92	94	0.24
PLP/N	1.79	46	63	73	80	94	95	0.03
				AD				
Random	4.15	10	26	35	39	54	58	0.37
R-L	4.14	10	25	36	43	61	66	3.76
Native	3.25	23	41	49	57	72	74	0.39
				**CSKDE212**				
				AC				
S/R	1.27	61	75	81	85	98	99	5.22
S/N	1.09	66	80	85	87	99	100	5.41
				GOLD				
PLP/R	1.96	39	60	71	78	91	91	0.03
PLP/N	1.62	48	67	76	83	95	96	0.03
				AD				
Random	3.78	13	30	40	45	61	65	0.36
Native	3.09	28	47	56	60	76	76	0.38

Table S8: Analysis of AC, AD, and GOLD docking failures on the CSKDE304 set. #SolvExp: number of cases with buriedness < 0.85; #CC: number of cases with crystal contacts; #LowEDIAm: number of cases with ligand EDIAm value ≤ 0.4; #Flex: number of cases with ligand DOF > 10. Percentages are reported in parentheses and refer to the total number of complexes (304) for the column #Cases, but to the value in column #Cases for all other columns.

Algorithm	#Cases	#SolvExp	#CC	#LowEDIAm	#Flex
			CSKDE304 Set		
–	304 (100)	191 (63)	119 (40)	20 (7)	87 (29)
			All Failures		
AC	66 (21)	52 (79)	26 (39)	13 (20)	28 (42)
AD	193 (65)	144 (75)	68 (35)	28 (15)	82 (42)
GOLD	102 (34)	81 (79)	41 (40)	21 (21)	44 (43)
			Sampling Failures		
AC	45 (15)	35 (78)	16 (36)	9 (20)	20 (44)
AD	184 (61)	136 (74)	63 (34)	25 (14)	75 (41)
GOLD	64 (21)	49 (77)	26 (41)	14 (22)	27 (42)
			Scoring Failures		
AC	21 (7)	17 (81)	10 (48)	4 (19)	8 (38)
AD	9 (3)	8 (89)	5 (56)	3 (33)	7 (78)
GOLD	38 (13)	32 (84)	15 (39)	7 (18)	17 (45)

Table S9: AC and GOLD re-docking and cross-docking results on the SARS-MP-76 and SARS-MP-39 sets. Sampling parameters (SP); median RMSD value of the best pose (RMSD, [Å]); success rate [%] of the best pose RMSD ≤ 1.0 Å (Best-1.0), 1.5 Å (Best-1.5) and 2.0 Å (Best-2.0); success rate [%] of best cluster (Cluster1), best five clusters (Cluster1-5), and in all final poses (All) with an RMSD ≤ 2.0 Å; median CPU time (Time, [h]). AC docking conditions are specified as N_{Thr}/method Graphical representation of the results for AC are presented in Fig. S10.

SP	RMSD	Best-1.0	Best-1.5	Best-2.0	Cluster1	Cluster1-5	All	Time
**SARS-MP-76**								
AC								
50/cross/rigid	4.17	3	11	26	30	54	80	3.43
50/re/rigid	1.61	34	50	59	67	86	91	3.44
60/cross/flex	4.19	3	12	25	30	53	68	5.91
60/cross/cys-flex	4.25	5	20	26	33	54	74	2.56
60/cross/rigid	4.28	3	11	28	30	54	82	1.91
60/re/rigid	1.71	26	49	58	67	84	92	1.90
70/cross/rigid	4.23	1	11	22	26	54	80	0.88
70/re/rigid	1.52	30	51	58	63	82	89	0.86
GOLD								
PLP/cross	6.16	3	7	17	26	47	51	0.01
PLP/re	2.58	21	34	45	49	80	80	0.01
**SARS-MP-39**								
AC								
60/cross/flex	3.14	3	18	36	44	74	90	5.22
60/cross/cys-flex	3.82	8	26	33	46	74	95	2.24
60/cross/rigid	3.98	3	13	33	38	77	92	1.66
60/re/rigid	1.33	33	56	64	69	82	90	1.81
GOLD								
PLP/cross	5.66	0	5	15	28	54	56	0.01
PLP/re	1.96	21	33	51	51	87	87	0.01

Supporting Figures

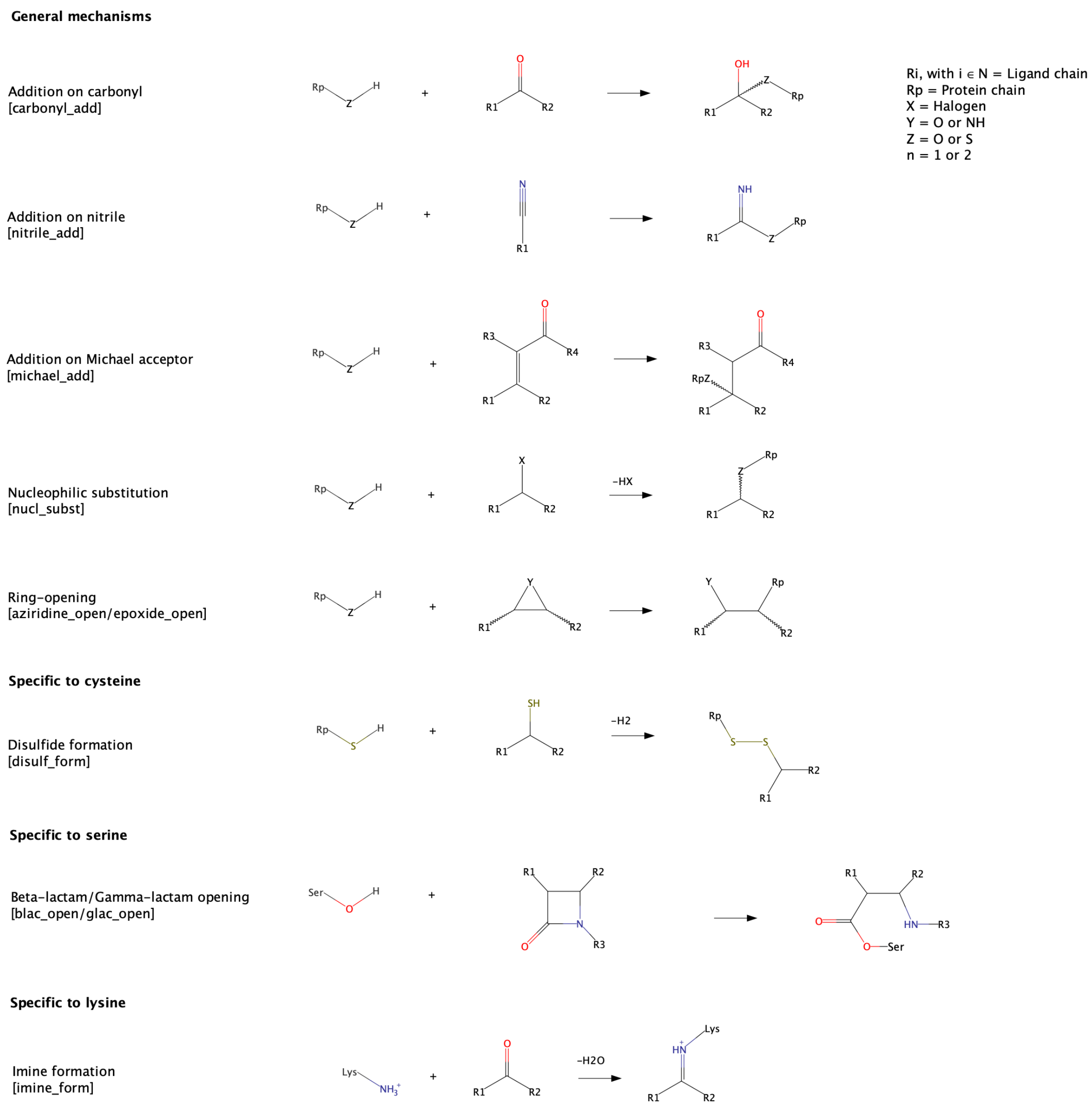

Figure S5: Summary of identified chemical reactions (with corresponding abbreviations) between ligands and amino acid side chains found in the PDB and included in the CSKDE95 set. Corresponding abbreviations are in brackets. Figure taken from Supporting Information of Ref 4.

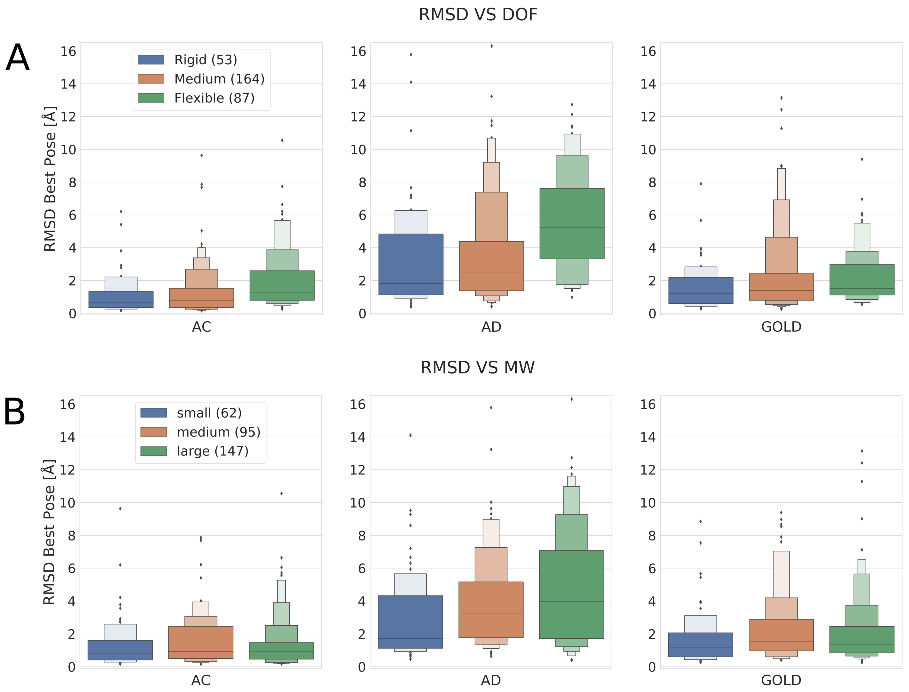

Figure S6: Best pose RMSD analysis of re-docking results obtained with AC, AD, and GOLD on the CSKDE304 set. Ligands are classified according to A. DOF: rigid (DOF < 5), medium ($5 \leq$ DOF ≤ 10) and flexible ($10 <$ DOF); B. Molecular weight (MW): small (MW < 300), medium ($300 \leq$ MW ≤ 400) and large ($400 <$ MW).

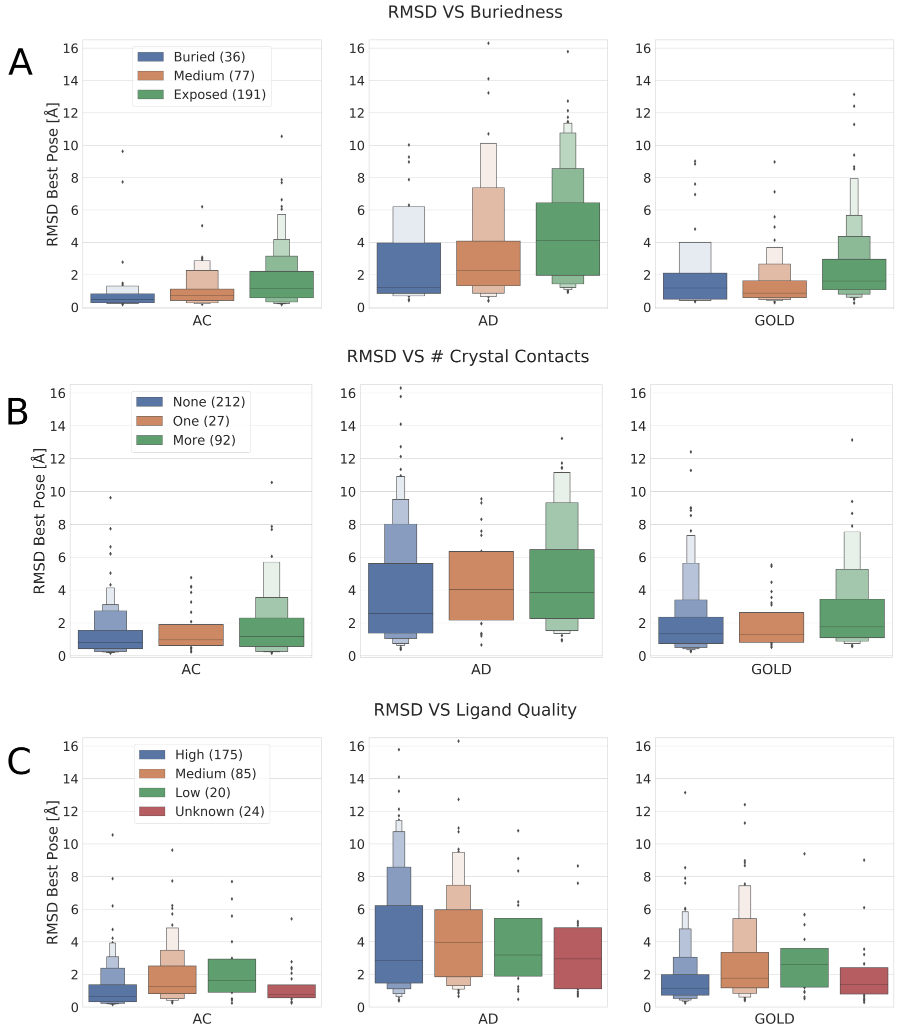

Figure S7: Best pose RMSD analysis of re-docking results obtained with AC, AD, and GOLD on the CSKDE304 set. Ligands are classified according to A. Buriedness: exposed (buriedness $< 0.85\%$), medium ($0.85 \leq$ buriedness ≤ 0.95), and buried ($0.95 <$ buriedness); B. Crystal contacts: None, one and more than one. C. Ligand quality: low(EDIAm ≤ 0.4), medium ($0.4 <$ EDIAm < 0.8), high ($0.8 \leq$ EDIAm) and unknown (could not be calculated).

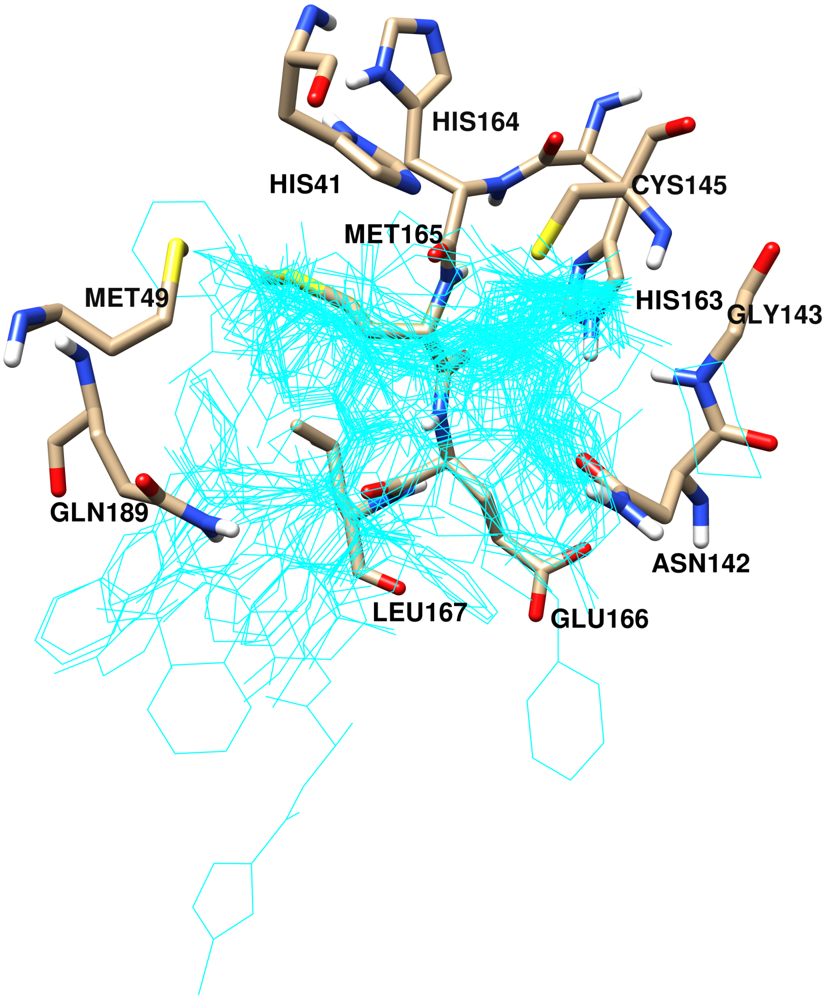

Figure S8: Native SARS-CoV-2 main protease ligand structures (cyan) after protein superimposition to target structure (PDB ID 7c7p). All protein residues clashing with at least one ligand are displayed, namely His41, Met49, Asn142, Gly143, Cys145, His163, His164, Met165, Glu166, Leu167, and Gln189. These residues were selected to be flexible in the flexible cross-docking runs.

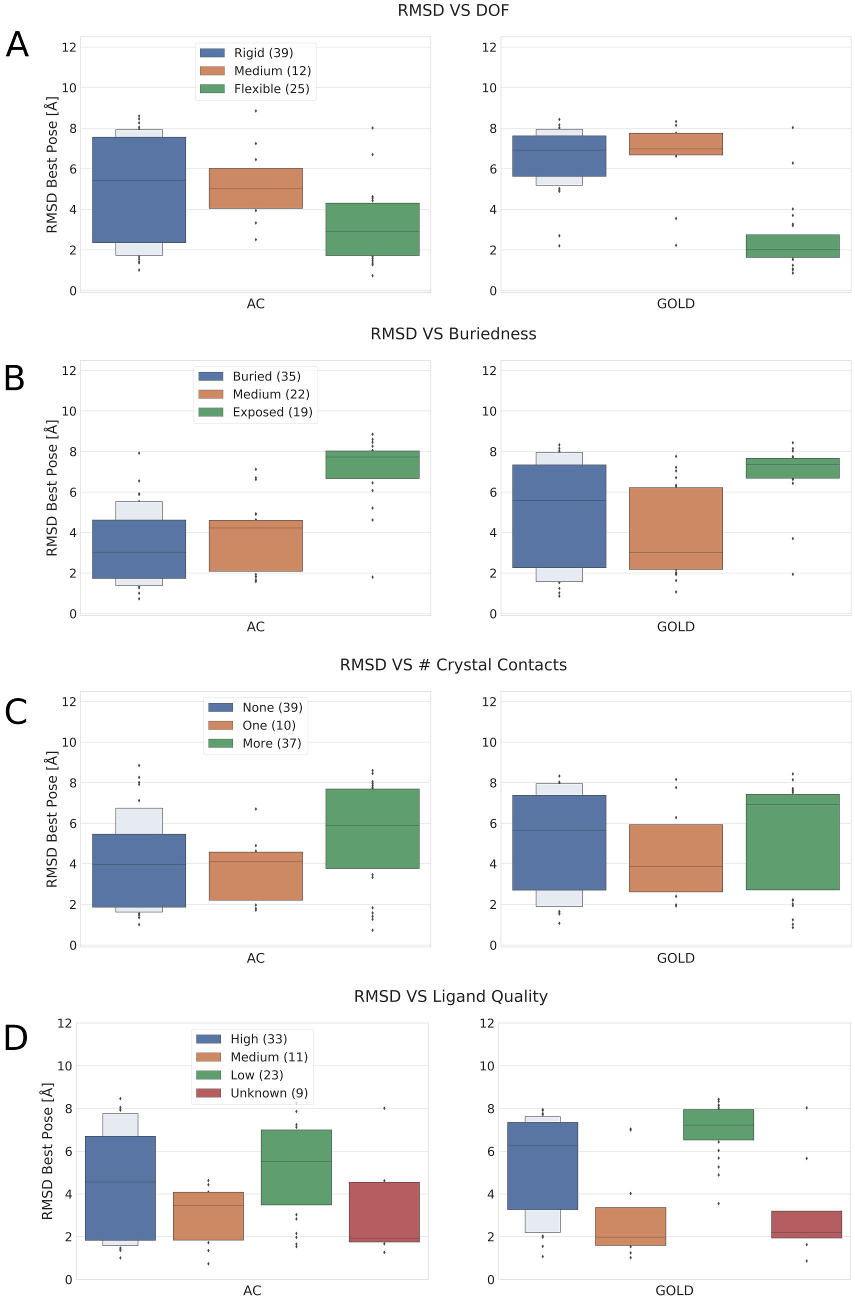

Figure S9: Best pose RMSD analysis of cross-docking results obtained with AC and GOLD on the SARS-MP-76 set. Ligands are classified according to : A. DOF: rigid (DOF < 5), medium ($5 \leq$ DOF ≤ 10) and flexible ($10 <$ DOF); B. Buriedness: exposed (buriedness $<$ 0.65%), medium ($0.65 \leq$ buriedness ≤ 0.75), and buried ($0.75 <$ buriedness); C. Crystal contacts: None, one and more than one. D. Ligand quality: low (EDIAm ≤ 0.4), medium ($0.4 <$ EDIAm < 0.8), high ($0.8 \leq$ EDIAm) and unknown (could not be calculated).

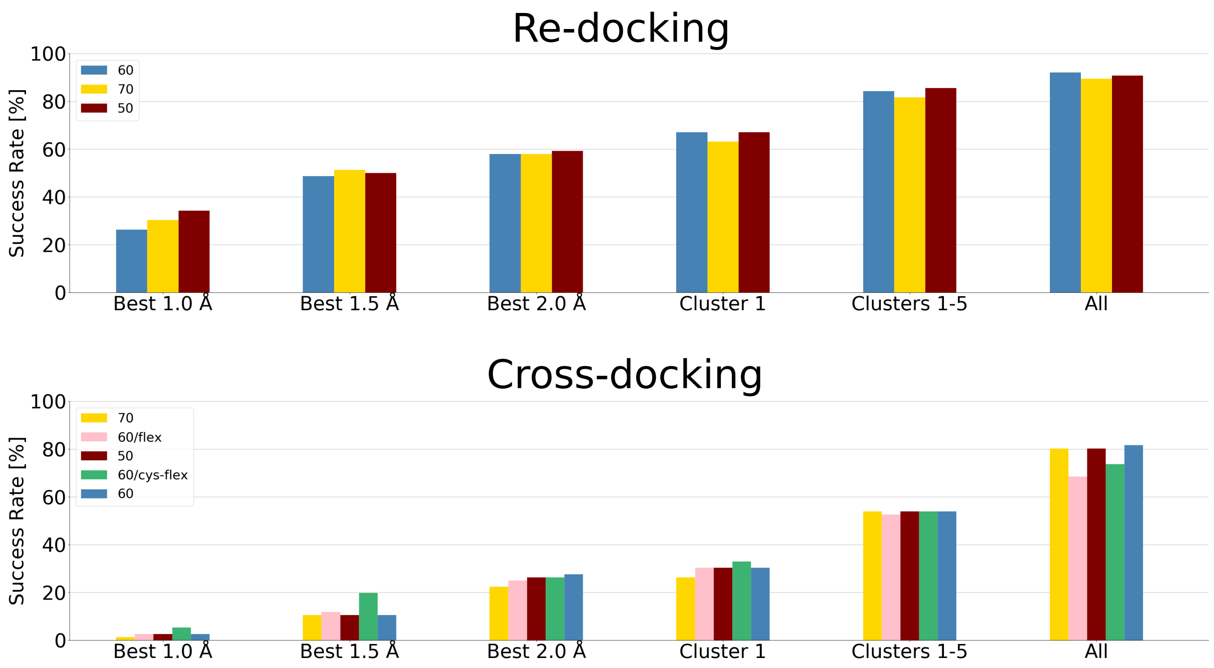

Figure S10: AC re-docking (A) and cross-docking (B) success rates on SARS-MP-76 set using different conditions. AC docking conditions are specified as N_{Thr}/method, with method = re (re-docking), cross (cross-docking), rigid (rigid protein), flex (flexible protein) and cys-flex (only the reactive Cys145 flexible). Numerical values given in Table S9.

References

(1) The UniProt Consortium, UniProt: the universal protein knowledgebase in 2021. *Nucleic Acids Res.* **2021**, *49*, D480–D489.

(2) Douangamath, A.; Fearon, D.; Gehrtz, P.; Krojer, T.; Lukacik, P.; Owen, C. D.; Resnick, E.; Strain-Damerell, C.; Aimon, A.; Ábrányi Balogh, P.; Brandão-Neto, J.; Carbery, A.; Davison, G.; Dias, A.; Downes, T. D.; Dunnett, L.; Fairhead, M.; Firth, J. D.; Jones, S. P.; Keeley, A.; Keserü, G. M.; Klein, H. F.; Martin, M. P.; Noble, M. E. M.; O'Brien, P.; Powell, A.; Reddi, R. N.; Skyner, R.; Snee, M.; Waring, M. J.; Wild, C.; London, N.; von Delft, F.; Walsh, M. A. Crystallographic and Electrophilic Fragment Screening of the SARS-CoV-2 Main Protease. *Nat. Commun.* **2020**, *11*, 5047.

(3) Hu, Q.; Xiong, Y.; Zhu, G.-H.; Zhang, Y.-N.; Zhang, Y.-W.; Huang, P.; Ge, G.-B. The SARS-CoV-2 main protease (Mpro): Structure, function, and emerging therapies for COVID-19. *MedComm* **2022**, *3*, e151.

(4) Bugnon, M.; Goullieux, M.; Röhrig, U. F.; Perez, M. A. S.; Daina, A.; Michielin, O.; Zoete, V. SwissParam 2023: A Modern Web-Based Tool for Efficient Small Molecule Parametrization. *J. Chem. Inf. Model.* **2023**, 10.1021/acs.jcim.3c01053.
